# A Scoping Review to Explore the Potential Benefits of Nutrition Interventions for Latino/a Adult Cancer Survivors in the US

**DOI:** 10.3390/nu15234963

**Published:** 2023-11-29

**Authors:** Cassandra M. Johnson, Emily Stubblefield, Brandon M. Godinich, Miranda Walker, Ramona Salcedo Price, Marlyn A. Allicock

**Affiliations:** 1 Nutrition and Foods Program, School of Family and Consumer Sciences, Texas State University, 601 University Drive, San Marcos, TX 78666, USA; ujf9@txstate.edu (E.S.); mw1431@txstate.edu (M.W.); rsp44@txstate.edu (R.S.P.); 2 Center for Health Promotion and Prevention Research and Center for Pediatric Population Health, Department of Health Promotion and Behavioral Sciences, The University of Texas Health Science Center at Houston School of Public Health, 2777 North Stemmons Freeway, Dallas, TX 75207, USA; brandon.m.godinich@uth.tmc.edu (B.M.G.); marlyn.a.allicock@uth.tmc.edu (M.A.A.); 3 Department of Medical Education, Paul L. Foster School of Medicine, Texas Tech University Health Sciences Center El Paso, 5001 El Paso Ave, El Paso, TX 79905, USA

**Keywords:** Latino cancer survivors, cancer survivorship, experimental designs, nutrition intervention, cancer disparity, cancer equity

## Abstract

Despite evidence for the role of healthy diets in preventing cancer, little is known about how nutrition can support positive health outcomes after a cancer diagnosis for Latino/a cancer survivors in the United States (U.S.). The purpose of this scoping review is to understand the potential benefits of nutrition interventions in supporting healthy survivorship among Latino/a cancer survivors in the U.S. A team compiled, evaluated, and summarized the available evidence. Potentially relevant studies were identified from a comprehensive search of peer-reviewed databases and the gray literature. Eligible studies included Latino/a adult cancer survivors with a nutrition education, dietary change, or behavioral intervention; and a nutrition-related health outcome. Data were extracted and summarized using tables. The review included 10 randomized controlled trials, with samples or subsamples of Latino/a cancer survivors. Interventions mostly focused on breast cancer survivors. The results showed some evidence that dietary behaviors, like fruit and vegetable intake, were related to positive outcomes, like a decreased risk of cancer (through changes in DNA methylation), decreased risk breast cancer recurrence (through changes in inflammatory biomarkers), or improved perception of health status. The findings highlight a need for community-engaged and culturally relevant nutrition interventions for Latino/a adults, especially for rural communities; and innovative intervention approaches, including m/ehealth approaches with long-term follow-up.

## 1. Introduction

In the United States (U.S.), adults of Hispanic heritage are the nation’s second largest racial or ethnic group, representing 19% of the population [1]. In some states, including California and Texas, the Hispanic population is the largest racial/ethnic group [2]. This demographic further distinguishes itself by being the second-fastest growing racial/ethnic group within the U.S. [2]. The U.S. Census Bureau population projections have indicated extensive shifts in the racial/ethnic demographics in the next four decades, as the proportion of non-Hispanic White individuals is expected to fall below 50% and Hispanic individuals increase to 27.5% of the U.S. population in 2060 [3]. 

Among Hispanic and Latino adults, cancer is among the leading causes of death and attributed to 20% of deaths [4]. The Hispanic/Latino population is differentially exposed to risk factors linked to cancer, such as poverty [5], and limited access to health care from health insurance [6], and this population is more vulnerable to experiencing severe outcomes, including mortality from cancer [4]. Despite having lower cancer rates than White individuals in the U.S, Hispanics/Latino individuals are disproportionately affected by certain types of cancer [4,7]. For instance, stomach, liver, and cervical cancer are more prevalent among Hispanics/Latinos [4,7]. Prior research also has shown that Hispanic/Latino patients with cancer are more likely to receive a late-stage or more advanced cancer diagnosis and more aggressive treatments compared to non-Hispanic/Latino patients [4,8], leading to higher mortality rates for stomach, liver, and cervical cancer [9]. The National Institute of Minority Health and Health Disparities (NIMHD) has identified “health determinants” as the root cause of cancer disparities [10]. For example, socioeconomic status (measured by income and education) and structural racism affect health and quality of life. Lower socioeconomic status is rooted in structural racism, leading to recurrent health and health care access disadvantages [11]. Health determinants for cancer include, but are not limited to, socioeconomic status, structural racism; access to and quality of health care (e.g., screening and treatment) [4]; lack of trust in health provider and patient–provider communication challenges [12]; environmental toxins and infectious agents (e.g., aflatoxin [13]); cultural values and beliefs; and acculturation, influence cancer occurrence and outcomes among Latino/a adults [4]. 

Advances and treatments in cancer survivorship have led to a growing number of cancer survivors and attention to improve quality of life. Since 1975, the number of cancer survivors has more than quadrupled and is expected to reach about 26 million people in 2040 [14]. The most recent estimates from 2022 indicate that more than 18 million people in the U.S. are cancer survivors [15]. Breast and prostate cancer represent the largest share of cancer survivors [15]. As the number of cancer survivors increases, there is increased interest in supportive care, including psychosocial and palliative care, to ensure the well-being and quality of life of cancer survivors and their caregivers [15]. 

Healthy behaviors can positively affect functioning and quality of life among cancer survivors [16]. In general, scientific, clinical, and patient advocacy communities have stressed the value of post-diagnosis lifestyle changes on cancer outcomes and diet as a key aspect of behavioral changes. The American Cancer Society (ACS) and the American Institute of Cancer Research (AICR) have issued guidelines recommending nutrition changes to reduce the risk of developing or dying from cancer; specifically, cancer guidelines recommend healthy dietary patterns that include high-nutrient foods and a variety of vegetables, fruits, and whole grains to promote favorable cancer outcomes [17,18,19]. However, guidelines have focused primarily on cancer prevention. 

Despite evidence for the role of healthy diets in preventing cancer [20,21], little is known about the role of nutrition in reducing negative health outcomes (e.g., cancer recurrence) or supporting positive health outcomes (e.g., well-being or quality of life) during survivorship, that is, following a cancer diagnosis for Hispanic and Latino/a adults (henceforth described as Latino/a cancer survivors) living in the U.S. Previously, scholars have conducted reviews of nutrition and physical activity interventions across the cancer continuum. However, their review papers were neither inclusive in terms of gender or cancer type nor focused on the post-diagnosis or survivorship and included interventions with relatively few Latino/a cancer survivors [22,23,24]. Specifically, this scoping review applied a systematic scoping approach to compile and synthesize more of the available evidence for Latino/a cancer survivors and answered the research question: what are the potential benefits of nutrition interventions in supporting healthy survivorship among Latino/a adult cancer survivors in the U.S.? Findings from this review will inform future research by identifying key gaps in the literature and motivating additional studies, guide practice by synthesizing evidence for best practices, and identify potential policy priorities for advancing equity in cancer survivorship among Latino/a adults.

## 2. Methods

### 2.1. Research Team

The research team included nutrition graduate students (ES and MW), a medical and public health student (BMG), and faculty members (CMJ and RSP) in nutrition, who specialize in nutrition interventions with Latino/a populations and nutrition and cancer, respectively, and a faculty member in behavioral science (MAA), who specializes in behavior change interventions for cancer survivors and cancer disparities.

### 2.2. Procedures

The team followed guidance outlined in the Preferred Reporting Items for Systematic Reviews and Meta-Analyses Extension for Scoping Reviews (PRISMA-ScR) [25], and scoping review methodology outlined originally by Arksey and O’Malley [26], adapted by Levac et al. [27], enhanced by Westphaln et al. in 2021 [28], and applied to a review of lifestyle interventions for adult cancer survivors [29]. Based on the Arksey and O’Malley Framework, we followed the six stages for conducting a scoping review: (1) specification of the research question; (2) identification of relevant literature; (3) selection of studies; (4) mapping the data (also called extraction); (5) summarization, synthesis, and report creation; and (6) inclusion of an expert consultation [26]. This scoping review answered the following research question: what is known about the potential benefits of nutrition interventions in supporting healthy survivorship among Latino/a cancer survivors?

*Literature search strategy*—To identify relevant literature for the U.S., this review completed a broad search of the literature, using two databases of peer-reviewed literature (PubMed/MEDLINE, CINAHL), as well as ProQuest Dissertation and Theses Global for doctoral dissertations. Searches were based on a combination of keywords for the following concepts: (1) Hispanic or Latino (e.g., Hispanic, Hispanic, Americans, Latin, and Latino/a); (2) cancer survivors (e.g., cancer survivors, cancer survivorship, and cancer survival); (3) cancer outcomes (e.g., cancer prognosis, neoplasms, and cancer outcomes); and (4) nutrition or diet (e.g., diet, diet therapy, dietary, food, and nutrition). The literature search captured records as of 28 January 2023, and no additional date was used to restrict the search. The exact search strategy used for PubMed/MEDLINE was (“Hispanic Americans” [mesh] or “Latino” [tiab] or “Latina” [tiab] or “Mexican” [tiab] or “Latin” [tiab] or “Latinx” [tiab] or “Hispano” [tiab]) AND (“survivorship” [mesh] or “cancer survivors” [mesh] or “survivors” [mesh] or “cancer survivorship” [tiab] or “cancer survival” [tiab] or “survival” [tiab] or “cancer prognosis” [tiab] or “neoplasms” [mesh] or “cancer outcomes” [tiab]) AND (“diet” [mesh] or “diet therapy” [mesh] or “nutrition therapy” [mesh] or “diet, food, and nutrition” [mesh] or “dietary” [tiab] or “nutrition” [tiab] or “nutritional status” [mesh]). Secondary sources included websites for the Cochrane Central Register of Controlled Trials (CENTRAL), ClinicalTrials.gov, and Reporter.NIH.gov assessed on 28 September 2023 [30,31,32]. A research assistant (RA) marked trials that were recruiting or not yet recruiting for additional follow-up. Supplementary Table S1 provides more details on secondary sources. 

Given the limited number of studies of nutrition and cancer for this priority population (Latino/a cancer survivors), the team connected with subject matter experts to identify any additional interventions and potentially unpublished research studies and obtain additional insights from the experts’ perspectives through consultation [27]. We created a list of subject matter experts based on our perception of leadership in the field, such as high-profile speakers or panelists at national events, including the National Institutes of Health (NIH) Pathways to Prevention Program workshop from July 2022 [33]; and highly cited investigators specializing in behavioral nutrition and cancer or cancer with Latino/a populations. The lead author (CMJ) corresponded with subject matter experts for assistance in identifying potentially eligible studies, including studies conducted in states with large populations of Latino residents, such as Texas and California. Emails to experts were sent in the spring of 2023. 

*Study selection*—The team selected relevant studies through a two-phase screening process conducted by a lead screener and assistant screener. For the initial screening, two trained research assistants (RAs) independently screened titles and abstracts to identify experimental studies related to the topic of nutrition and cancer with the priority population of Latino/a adults with cancer or Latina/o/e cancer survivors. RAs discussed discrepancies with the lead author. To pass initial screening, studies had to be related to nutrition or diet with a sample of Latino/a adult cancer survivors or a racially/ethnically diverse sample that included Latino/a adult cancer survivors. When the information from the title and abstract were insufficient to determine relevance, the RAs reviewed the full-text version of the article or report. All records that met initial screening requirements were retained for the secondary screening. 

**Figure 1:**
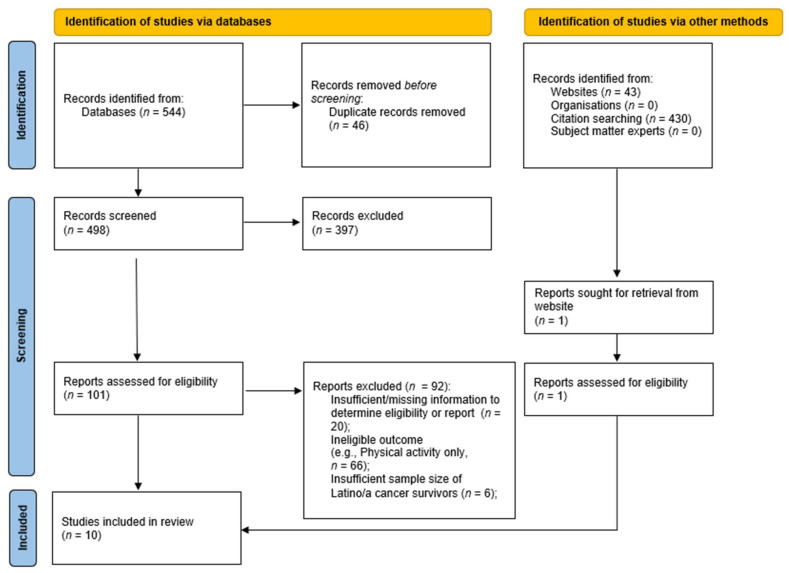
PRISMA flowchart for identification, screening, and eligibility determination. This figure shows the number of records (*n*) at each stage of the scoping review. This figure is based on the PRISMA flowchart [35]. Databases included PubMed/MEDLINE, CINAHL, and ProQuest Theses and Dissertations Global. Websites included ClinicalTrials.gov, Cochrane CENTRAL, and Reporter.NIH.gov. Supplementary Table S1 provides additional details on the websites. PRISMA: Preferred Reporting Items for Systematic Reviews and Meta-Analyses.

For the secondary screening, the lead screener evaluated each record based on eligibility criteria, defined based on PICOS, where “P” stands for population, “I” is intervention or exposure, “C” is comparison, “O” is outcome, and “S” is study design [25,34]. Supplementary Table S2 shows how the PICOS components were operationalized for this review. The lead author reviewed screening decisions and discussed decisions with the lead screener. In addition, a second screener reviewed screening decisions. When needed, the lead author (CMJ) emailed the corresponding authors and co-authors of potentially eligible studies to obtain information required to determine eligibility. For example, emails were sent to confirm the sample size of the Latino/a subgroup of the total sample and whether they had completed subgroup analyses for Latino/a adults, had analyzed intervention effects (for rationale or design articles that did not report results or trials identified using websites), or had completed post hoc analyses of interventions. This review excluded any study where there was insufficient information to determine eligibility or when corresponding authors could not confirm or clarify details needed for eligibility determination. The screening is documented in Figure 1 [35].

Only experimental designs with human participants were eligible. This review included randomized controlled trials (RCTs) and quasi-experimental designs that did not use random assignment. Nutrition interventions or programs included nutrition education, dietary modification or dietary change component, or behavioral nutrition. This review excluded non-experimental designs, such as protocols (intervention description without results), cross-sectional and longitudinal studies, qualitative studies, reviews or commentaries, perspectives, or editorials. Relevant protocols for RCTs were marked for follow-up. The lead author inquired about additional publications with results for RCTs. In addition, other relevant studies were used to create a list of potential subject matter experts and develop the discussion section of this manuscript. Relevant reviews or meta-analyses, published in the last five years were retained, and their reference lists were scanned to identify potentially eligible interventions. No additional articles were added from the reference review. RAs made notes about interventions that had been terminated or were in the design or recruitment phase (see Supplementary Table S3 for future trials). 

Interventions were eligible for inclusion if they reported on a nutrition intervention, as defined previously, for adult Latino/a cancer survivors (100% sample Latino/a adults), or interventions that included a Latino/a subgroup (*n* ≥ 20 Latino/a participants or a Latino/a subgroup of ≥15% of the total sample). Previous reviews have included samples with 18 to 20 participants in total [22,24]. Larger studies with a relatively low proportion of Latino/a adults (below 15%) were eligible because the sample size of the Latino/a subgroup was at least 20 participants. Intervention participants had to be adult cancer survivors, that is, diagnosed with cancer as an adult, defined as 18 years or older. Participants included cancer survivors who had already completed active treatment or were currently in active treatment with or without endocrine therapy. The 15% cutoff for the proportion of Latino/a participants was designed to be conservative and enable more potentially eligible interventions to be included. In 2019, Hispanic and Latino/a individuals were 19% of the U.S. population based on U.S. Census 2022 data [1]. There were no requirements for the type of cancer, stage of cancer at the time of diagnosis, or gender of the intervention participants.

This review included a range of nutrition-related outcomes. To be eligible for inclusion, studies reported outcomes related to, but not limited to, appetite, eating behaviors, dietary intake, weight, well-being or quality of life, indicators of chronic disease or adverse cardiometabolic health, or cancer recurrence. A broad range of outcomes was used to capture both negative and positive health outcomes related to nutrition and cancer. 

There were no criteria related to the publication type. In other words, a full-text, peer-reviewed article was not a requirement for inclusion. During the initial screening, doctoral dissertations and abstracts were marked for follow-up. Additional information was obtained from ClinicalTrials.org and Reporter.NIH.gov [30,31]. In addition, the lead author (CMJ) emailed the corresponding authors and co-authors of potentially eligible studies to obtain information through personal communication. 

*Data management*—Throughout the study identification and study selection process, the team used Excel workbooks to manage the literature search and study selection and used Word tables to manage the data extraction process. The lead screener (MW) exported all records from each database into a worksheet and removed any duplicates. The record titles were hyperlinked to the database entry to expedite screening. Then, she combined records from the databases into one worksheet and removed any duplicates. For the initial screening (title and abstract), two reviewers (MW and CL) independently screened all records. They used their own worksheets to document decisions, notes, or comments. The lead author (CMJ) reviewed the results of the primary screening and helped resolve any disagreements. All records marked as a “yes” were retained for the secondary screening.

For the secondary screening, the lead screener (MW) created a new worksheet and used this worksheet to mark decisions related to the full-text review. When the information in the full-text version was not clear or sufficient to determine eligibility, the lead screener (MW) discussed the study with the lead author (CMJ), and they referenced additional information from ClinicalTrials.gov or Reporter.NIH.gov [30,31], or personal communication with corresponding authors of published studies, as needed. A second reviewer (ES) checked the secondary screening to consider bias in screening. 

The lead screener documented comments about eligibility and notes to discuss (e.g., subgroup analyses, available results for intervention/program effects, or post hoc analyses). For example, if a study was potentially eligible but no results were available (e.g., intervention description or baseline characteristics only), then the lead author (CMJ) emailed the corresponding author of the study to inquire. Secondary reviewers (CL and ES) helped with the secondary screening. Screening decisions were discussed with the lead author (CMJ), who also checked decisions. Figure 1 presents the PRISMA flowchart and documents the flow of records from the initial literature search through study selection and inclusion in this scoping review [35].

### 2.3. Data Extraction of Included Studies

Two reviewers (ES and BNJ) extracted data from the included studies into Word tables. Information was placed into tables for data extraction. The reviewers extracted general information about the intervention, including setting and sample, and details related to intervention development (e.g., theory used), implementation (e.g., delivery mode and dose), and evaluation (e.g., primary outcomes and secondary outcomes). Reviewers also documented their comments in a separate and unpublished notes column. Each reviewer completed extraction for half of the studies and reviewed the other half for accuracy. Any issues or questions were discussed with the lead author. As a team, we reviewed related publications for that intervention and websites (e.g., ClinicalTrials.gov, Reporter.NIH.gov). If reviewers did not find the required information for data extraction from available sources, the lead author used personal communication with the corresponding author or co-authors of the study of interest. Personal communication was used to obtain results for one study.

### 2.4. Summarization and Synthesis of Research Findings

The lead author (CMJ) summarized and synthesized information from the included interventions in narrative form, and the co-authors contributed by reviewing and developing the summary and synthesis. Key messages for translational research were developed in consultation with experts [27].

## 3. Results

### 3.1. Overview

Figure 1 shows the PRISMA flowchart of records through screening, eligibility determination, and inclusion. Potential records were identified through databases (*n* = 544), of which 46 were duplicates. Of the 498 records screened, 101 were retained for eligibility determination. Records were excluded for reasons, such as interventions focused only on physical activity, or insufficient sample size of Latino/a cancer survivors (*n* = 92). Nine interventions were eligible from the databases. Additional records from clinical trial websites were screened (*n* = 43) to identify recent interventions, and one new intervention was added. In total, ten nutrition interventions, all RCTs conducted in the U.S., were included in this scoping review (Table 1): Avanzando Juntas [36], Bronx Oncology Living Daily (BOLD) Healthy Living [37], Cocinar para Su Salud [38,39,40], La Vida Activa [41], LIVES (Lifestyle Intervention for Ovarian Cancer Enhanced Survival) [42,43], Mi Vida Saludable [44,45,46], My Health [47], Nuestra Salud [48], Prescription (Rx) for Better Breast Health [49,50], and WHEL (Women’s Healthy Eating and Living Study) [51,52]. Ongoing or future planned trials and terminated trials are shown in Supplementary Table S3. 

### 3.2. Study Design

**Figure 2:**
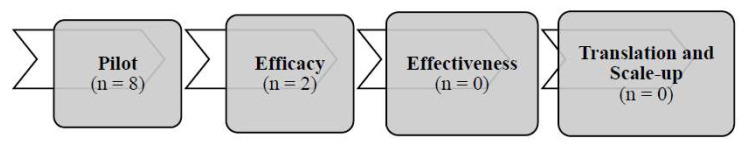
Number of studies across the intervention and implementation science continuum. This figure shows the number of studies by intervention type for the 10 studies included in this scoping review. Preclinical included feasibility or pilot trials. Eight of the studies were designated as pilots. There were two studies designated as efficacy trials, the Women’s Healthy Eating and Living study (WHEL [51,52]) and Mi Vida Saludable [44,45,46]. There were no studies designated as effectiveness trials.

Eight studies were classified as feasibility or pilot studies, including five author-defined pilot RCTs, namely the Avanzando Juntas [36], BOLD [37], La Vida Activa [41], Nuestra Salud [48], and Prescription (Rx) for Better Breast Health [49,50]. Figure 2 shows the number of studies across the continuum, including preclinical, clinical, and translation and implementation trials. Two studies were determined to be efficacy trials (Women’s Healthy Eating and Living or WHEL study) [51,52] and Mi Vida Saludable [44,45,46]), based on having a previous feasibility or pilot study published (WHEL pilot [53] and Cocinar para Su Salud [38,39,40]). The WHEL pilot was not considered for this review [53], because no data were reported on race or ethnicity, likely due to the timing of the study in the 1990s, but this review included Cocinar para Su Salud [38,39,40], the pilot study for Mi Vida Saludable [44,45,46].

### 3.3. Intervention Foci

The studies included interventions focused on lifestyle or health promotion, weight loss, and chronic disease prevention (e.g., diabetes prevention), and two focused explicitly on cancer-free progression (see Table 1). Three studies reported on culturally adapted or culturally based interventions (Avanzando Juntas [36], Cocinar para Su Salud [38,39,40], and My Health [47]); these studies were also described as community-engaged or community-based interventions. In addition, one study Prescription (Rx) for Better Breast Health was a patient-navigated intervention [49,50].

### 3.4. Participant Characteristics

Included studies varied according to the sample size (Table 1). In studies with only Latino/a participants (100% sample), the number of Latino/a cancer survivors ranged from 37 to 167 adults. For studies with mixed samples, defined by Latino/a and non-Latino/a participants, the total number of participants ranged from 42 to 1205, represented 5.3% to 51.2% of the total sample size. The studies encompassed a diverse range of cancer survivor populations, representing varying cancer types. While two studies (BOLD [37] and Nuestra Salud [48]) examined survivorship across various cancer types, and one study focused on two types of cancer (Avanzando Juntas [36]), most studies concentrated on a specific type, such as breast cancer (*n* =7). Most studies (*n* = 9) restricted eligibility to survivors of early-stage cancer (0-III). Nine studies examined cancer survivorship in individuals who had completed active treatment at least two months prior to enrollment. One study, Nuestra Salud [48], included individuals who were undergoing active treatment.

### 3.5. Settings

All studies were conducted within the U.S. (Table 1), though there was one multisite study that recruited across the U.S. and Canada. In terms of location, four studies were carried out in the Northeastern region of the U.S. (e.g., New York). Two studies were completed in the Midwestern region (e.g., Illinois and Wisconsin), and two studies were conducted in the South or Southwestern region (e.g., Arizona and Texas). There were two multisite studies that recruited from sites across the U.S. or the U.S. and Canada. Importantly, all studies recruited primarily from large metropolitan or urban areas. Intervention sites varied across studies: remote-only studies completed primarily via telephone or internet (*n* = 3, LIVES [42,43], My Health [47], and Nuestra Salud [48]), clinical (*n* = 1, Mi Vida Saludable [44,45,46]), or community (*n* = 3, Avanzando Juntas [36], BOLD [37], and La Activa Vida [41]) sites (Table 1). Three studies had more than one site (Mi Vida Saludable [44,45,46], Prescription (Rx) for Better Breast Health [49,50], and WHEL [51,52]). 

### 3.6. Approaches and Theory 

Four studies described explicitly community-engaged or -based approaches in intervention design, implementation, or evaluation, such as working with community partners in an iterative process (BOLD [37], Cocinar para Su Salud [38,39,40], Mi Vida Saludable [44,45,46], and My Health [47]). One study reported being a community-situated study, because the Curves Weight Management program was accessible and available within the community (La Activa Vida) [41]. Most studies (*n* = 8) utilized a theory, model, or conceptual framework in intervention design, implementation, or evaluation, and two studies did not report on the use of a theory (Table 1). Notably, Social Cognitive Theory (SCT) was used in four of the studies: Cocinar para Su Salud [38,39,40], LIVES [42,43], Mi Vida Saludable [44,45,46], Nuestra Salud [48], and Prescription (Rx) for Better Breast Health [49,50]. Interventions informed by SCT integrated activities targeting key constructs, such as self-efficacy, self-monitoring, and self-regulation; skills building; or observational learning and modeling. Two studies, Cocinar para Su Salud [38,39,40] and Mi Vida Saludable [44,45,46], integrated the Transtheoretical Model (TTM). The TTM focuses on the stages of change when adopting new behaviors, including dietary modifications. Cocinar para Su Salud applied the TTM by focusing on pros and cons of change and self-efficacy in group discussions [38,39,40]; Prescription (Rx) for Better Breast Health applied stages of change by tailoring newsletters based on readiness for change [49,50].

### 3.7. Intervention Components, Curricula, and Behavior Change Strategies

The most common intervention components were nutrition education, cooking classes or workshops, or tele-coaching sessions. One intervention tested a commercial weight loss class with the Curves Weight Management Program for Latina cancer survivors and included Curves curriculum, one-on-one and group exercises classes, and group nutrition classes for intervention participants (La Vida Activa) [41]. Some interventions had components such as a food-shopping field trip (Cocinar para Su Salud [38,39,40]). Four studies included motivational calls, including calls based on principles of motivational interviewing (MI) (LIVES [48], My Health [47], Nuestra Salud [48], and Prescription (Rx) for Better Breast Health [49,50]). Several studies (*n* = 6) mentioned the use of the national cancer-specific guidelines, such as the guidelines for diabetes prevention published by the American Diabetes Association (ADA) or cancer prevention published by American Cancer Society (ACS) and the American Institute for Cancer Research (AICR) for developing curricula or defining behavioral goals. Common nutritional goals included 4–5 servings per day of vegetables, particularly cruciferous and dark leafy green; 2–3 servings per day of fruits; dietary fiber intake of ≥30 g per day; and minimizing energy intake from fat to 15–20% daily (Cocinar para Su Salud, LIVES [38,39,40], Mi Vida Saludable [44,45,46], and WHEL [51,52]). Behavioral strategies included problem solving, goal setting, action planning, and self-monitoring (Cocinar para Su Salud, [38,39,40], My Health [47], and WHEL [51,52]). Regarding specific intervention components, curricula, or strategies for Latino/a cancer survivors, three studies incorporated culturally appropriate or relevant recipes (Cocinar para Su Salud [38,39,40], Mi Vida Saludable [44,45,46], and My Health [47]). 

### 3.8. Delivery and Dose

Studies were quite different in delivery approaches, including the format, training, and interventionists (Table 2). In-person or physically face-to-face delivery was more common compared to interventions delivered remotely or virtually; three studies were remote only (LIVES [42,43], My Health [47], and Nuestra Salud [48]). One study leveraged digital or technology for intervention delivery through a device-based application (My Health [47]) and another was ehealth, using an interactive project website (Mi Vida Saludable [44,45,46]). Tele-coaching sessions were a key part of six studies (La Vida Activa [41], LIVES [42,43], My Health [47], Nuestra Salud [48], Prescription (Rx) for Better Breast Health [49,50], and WHEL [51,52]). Additionally, telephone calls or text messages or emails were used for behavioral reinforcements in two (Cocinar para Su Salud [38,39,40] and La Vida Activa41) and two (BOLD [37] and LIVES [42,43]) studies, respectively. 

Generally, interventionists were health coaches or counselors trained for the intervention. Four studies provided training in motivational interviewing to interventionists (LIVES [42,43], My Health [47], Prescription (Rx) for Better Breast Health [49,50], and WHEL [51,52]). 

Regarding language, most interventions had bilingual delivery with materials and activities in English and Spanish (*n* = 7). Half of the studies reported working with bilingual or bicultural interventionists (Cocinar para Su Salud [38,39,40], La Vida Activa [41], Mi Vida Saludable [44,45,46], My Health [47], and Nuestra Salud [48]). Two interventions were delivered exclusively in English (Prescription (Rx) for Better Breast Health [49,50] and WHEL [51,52]), and one intervention was delivered exclusively in Spanish (Cocinar para Su Salud [38,39,40]). Notably, one intervention Mi Vida Saludable (*n* = 1) was delivered by a bilingual, multicultural, and multidisciplinary team [44,45,46]. 

The number of contacts and duration varied across the studies (Table 2). The number of contacts ranged from 6 (La Vida Activa [41]) to 33 (LIVES [42,43]), and the active intervention period ranged from 4 weeks (1 month) to 12 months. Four interventions had relatively short durations of 12 weeks or less, including BOLD [37], Cocinar para Su Salud [38,39,40], My Health [47], and Nuestra Salud [48]. Four other interventions spanned between 3 and 6 months, including Avanzando Juntas [36] and Prescription (Rx) for Better Breast Health [49,50]. In contrast, four interventions lasted for 12 months: Cocinar para Su Salud [38,39,40], La Vida Activa [41], and Mi Vida Saludable [44,45,46]. Based on authors’ estimations of the doses delivered, studies ranged from a dose of 5 h to 48 h, though this review was unable to calculate dose for half of the studies (Table 2). The author reported a total dose for one study (Cocinar para Su Salud [38,39,40]).

### 3.9. Outcome Assessment

All studies assessed primary and secondary outcomes at pre-test or baseline and immediately post-test (see Table 3). Only four studies included a follow-up measure to assess outcomes in the short term (6–18 months) post-intervention (*n* = 3) (La Vida Activa [41], LIVES [42,43], and Prescription (Rx) for Better Breast Health [49,50]), or in the long term (5 years) after the intervention (*n* = 1) (WHEL [51,52]). Importantly, two studies used cancer recurrence or death [42,43,51,52]. Cancer recurrence was measured using different techniques, including tissue collection, histology report, radiology image, or measures of CA-125; and a cancer antigen, at or above two-fold normal values (LIVES [42,43] and WHEL [51,52]).

Interventions reported primary outcomes related to cancer recurrence (*n* = 2), dietary intake (e.g., fruit and vegetable intake), body composition or weight loss (*n* = 2), or inflammation (*n* = 1) (Table 3). No studies used well-being or quality of life as a primary outcome. Positive health outcomes, including well-being or quality of life, were measured as secondary outcomes in four studies (Avanzando Juntas [36], BOLD [37], Prescription (Rx) for Better Breast Health [49,50], and WHEL [51,52]). Across the studies, secondary outcomes included perceived health status (*n* = 1 studies: BOLD [37]); mental health (*n* = 4 studies), such as anxiety, depression, social isolation, and social support (Cocinar para Salud [38,39,40], Mi Vida Saludable [44,45,46], Prescription (Rx) for Better Breast Health [49,50], and WHEL [51,52]); and psychosocial factors related to behavior change (*n* = 4 studies), such as health behavior change, stages of change, or readiness for change for dietary modification, motivation, nutrition knowledge, or self-efficacy (BOLD [37], Cocinar para Su Salud [38,39,40], Nuestra Salud [48], and Prescription (Rx) for Better Breast Health [49,50]). Nuestra Salud, an integrated symptom management and lifestyle behavior intervention, also assessed symptoms, including shortness of breath, pain, sleep difficulties, bowel problems, nausea, vomiting, numbness or tingling, swelling in hands and feet, skin rashes or sores, difficulty concentrating, poor appetite, depression, anxiety, fatigue, and cough [48]. These outcomes are not shown in Table 3.

Generally, outcome evaluation was completed with varied data collection techniques, including surveys (e.g., Food Frequency Questionnaire), biomarkers (e.g., blood plasma carotenoids and inflammation indicators), and anthropometry (e.g., weight and waist circumference). Studies conducted dietary assessment with varied techniques, including 24-h dietary recalls (*n* =3) and a Food Frequency Questionnaire (FFQ) (*n* = 4). One study used a three-day food diary and 14-item Diet Assessment Tool (Prescription (Rx) for Better Breast Health [49,50]). Four studies used blood samples to determine carotenoids as a biomarker of dietary intake of FVs, in addition to dietary recalls (Cocinar para Su Salud [38,39,40], Mi Vida Saludable [44,45,46], and WHEL [51,52]) or an FFQ (LIVES [42,43]). Regarding quality-of-life assessments, some studies used brief measures (e.g., two items from the SF-36, the Short-Form Health Survey used in BOLD [37]), while others used the PROMIS (Patient-Reported Outcomes Measurement Information System) scale (Avanzando Juntas [36] and Mi Vida Saludable [44,45,46]). Seven studies collected data on biomarkers [36,38,41,42,44,49,51], including indicators of adverse cardiometabolic health and inflammation, such as serum lipid, HOMA-IR, and hs-CRP. Three studies evaluated blood carotenoid concentration (LIVES [42,43], Mi Vida Saludable [44,45,46], and WHEL [51,52]) as a biomarker of dietary intake of FVs (Table 4). Unfortunately, only three studies reported on biomarker outcomes (Cocinar para Su Salud [38,39,40], La Vida Activa [41], and WHEL [51,52]).

### 3.10. Intervention Effects

Half of the studies (*n* = 5) reported a statistically significant effect of the intervention on the primary outcome in the hypothesized direction (BOLD [37], Cocinar para Su Salud [38,39,40], La Vida Activa [41], My Health [47], and Nuestra Salud [48]), while the remaining studies reported a null or non-significant effect of the intervention on primary outcomes (Avanzando Juntas [36] and WHEL [51,52]). There were some studies that have not reported on primary outcomes, or the results were not available (LIVES [42,43], Mi Vida Saludable [44,45,46], and Prescription (Rx) for Better Breast Health [49,50]).

This section summarizes intervention effects for various outcomes, including cancer-related outcomes like recurrence, well-being and quality of life, diet, body composition or weight loss, and cardiometabolic and inflammation outcomes. Five studies evaluated intervention effects on physical activity (Avanzando Juntas [36], La Vida Activa [41], LIVES [42,43], Mi Vida Saludable [44,45,46], My Health [47], and Nuestra Salud [48]), but those effects are not discussed here. Table 3 presents the main and secondary effects for each intervention. Table 4 presents the effects for cardiometabolic and inflammation biomarkers. For cancer-free progression, severity of symptoms, or perceived pain, there were two studies that examined cancer-free progression as primary outcomes; one study had results available and did not find effects of a plant-based, high-fiber diet on cancer recurrence or mortality (WHEL) [51,52]. Regarding other cancer-related outcomes, two studies reported effects for reduced symptom severity, including perceived pain (BOLD [37] and Nuestra Salud [48]). BOLD, a community-based intervention, demonstrated borderline significant decreases in perceived pain as moderate/severe (45.5% to 38.2% at post-test; *p* = 0.05) [37]. Nuestra Salud, a lifestyle and symptom management intervention, reported medium-to-large effects for summed symptom severity for cancer survivors (*d* = 0.74) and small effects for global symptom distress survivors (*d* = 0.17) and self-efficacy for managing symptoms survivors (*d* = 0.01) [48]. Regarding well-being and quality of life, one study (BOLD) reported significant pre/post-improvements in perceived health as good/excellent (66.0% to 75.5%; *p* = 0.001) [37]. For effects on dietary outcomes, three studies demonstrated effects for decreased calories from fat or increased dietary fiber, fruit, vegetables, or combined fruits and vegetables (FVs) (Cocinar para Su Salud [38,39,40], My Health [47], Nuestra Salud [48], and WHEL [51,52]). Prescription (Rx) for Better Breast Health demonstrated the effects of a patient-navigated, anti-inflammatory intervention on improvements in Mediterranean diet score at six months [50]. In a subgroup analysis of WHEL participants by race and ethnicity, Hispanic cancer survivors in the intervention group demonstrated significant improvements in percent of calories from fat, dietary fiber, fruits, and vegetables versus those in the comparison group at post-test (one year) and follow-up (four years) [51]. Cocinar para Su Salud, a culturally based intervention, reported an increase in servings of all FVs and targeted FVs at 3 months, which correlated to increased carotenoids measured in the blood [40]; the study also reported effects on percent of calories from fat [38,39,40]. Notably, effects on all FVs and targeted FVs were maintained at six months [38,39,40].

In terms of body composition, two studies reported significant improvements in waist circumference (WC) at 12 weeks (BOLD [37]) or 6 months (Cocinar para Su Salud [38,39,40]). One study reported marginally statistically significant effects on WC and statistically significant effects on weight loss (La Vida Activa [41]). Among the intervention participants in the La Vida Activa trial, weight loss ≥ 5% resulted in increased IGFBP-1, regulator of pro-tumorigenic IGF-1, and decreased serum glucose. Specifically, fat loss ≥ 2% resulted in decreased insulin, glucose, and HOMA-IR at 6 months [41].

Regarding cardiometabolic and inflammation outcomes, there was little information on effects, because four (of the seven studies) did not analyze biomarker data or make the results available [36,42,46,50]. In Cocinar para Su Salud, participants demonstrated a shift towards incorporating more fruits, vegetables, and whole grains into their diets [40]. Due to the beneficial anti-inflammatory properties in fruits and vegetables, pro-inflammatory markers associated with progression and recurrence were measured, including IL-1α, IL-6, IL-8, IL-10, hs-CRP, GM-CSF, and TNF-α. Non-significant differences in inflammation-related biomarkers were observed in the intervention group [40]. Increased global DNA methylation is associated with genomic instability, a hallmark of cancer development and progression, while fruit and vegetable intake can promote genomic stability. Global DNA methylation was measured in the Cocinar para Su Salud study [40], and, though not significant, borderline increased global DNA methylation was observed among the participants in the intervention group (Table 4).

### 3.11. Retention and Engagement Strategies

There were differences in the retention and engagement strategies. Five studies described incentives to encourage participation through recruitment and retention and promote behavior change (Cocinar para Su Salud [38,39,40], LIVES [42,43], Mi Vida Saludable [44,45,46], Nuestra Salud [48], and WHEL [51,52]). For example, small items, like water bottles, insulated lunch bags, or measuring cups, were provided as cues to action or for positive reinforcement in the LIVES study [42,43]. Other studies provided kitchenware or exercise wear (Mi Vida Saludable [44,45,46]) or provided high-quality juicers and offered them to participants at a significant discount and created an incentive point system with a prize raffle (WHEL [51,52]). Several studies used periodic incentives or newsletters to promote retention, such as LIVES [42,43]. Some studies gave participants a FitBit, used for self-monitoring of physical activity, to keep (Mi Vida Saludable [44,45,46] and Nuestra Salud [48]) or used monthly telephone calls with the study coordinator to promote retention (Cocinar para Su Salud [38,39,40] and Mi Vida Saludable [44,45,46]).

### 3.12. Attrition and Attendance

Regarding loss to follow-up or attrition, studies reported losing between 9.5% and 37% of participants between the enrollment and post-test assessment (Supplementary Table S4). Some studies had relatively low loss to follow-up, such as La Vida Activa [41] with 9.5% at 12 months, LIVES [42,43] with 11.5% at 24 months, and Cocinar para Su Salud with 13% attrition at six months [38,39,40]. Attendance for group sessions or coaching sessions varied (Table S4). For example, 80% of cancer survivors completed 12 tele-coaching sessions in Nuestra Salud [48], but only 38% attended the nine sessions in Cocinar para Su Salud [38,39,40]. In addition, half of the studies (*n* = 5) reported measuring adherence (La Vida Activa [41], LIVES [42,43], Nuestra Salud [48]), Prescription (Rx) for Better Breast Health [49,50], and WHEL [51,52], Table S4). Generally, effects were associated with greater adherence to intervention targets for lifestyle behaviors, including nutrition targets (La Vida Activa [41], Prescription (Rx) for Better Breast Health [49,50], and WHEL [51,52]); however, in La Vida Activa, there were no statistically significant differences in change in adherence by ethnicity at 6 or 12 months [41].

## 4. Discussion

The current review examined interventions for Latino/a cancer survivors in the U.S. through January 2023 and extends what is known beyond previous reviews, which searched through August 2021, August 2022, and October 2022 [22,23,24]. Based on the authors’ knowledge, this scoping review is the first to report on nutrition interventions post-diagnosis, that is, through active treatment and post-treatment survivorship, specifically for Latino/a adults, regardless of cancer type or gender. Only one study (Nuestra Salud) included participants in active treatment (*n* = 6 out of 37 cancer survivors) [48]. Findings from this review relate to Latino/a adults in the U.S. who are at least two months post-treatment.

Eight RCT studies provided evidence of potential benefits of nutrition interventions for Latino/a cancer survivors, including increases in perceived health status and symptom severity; improvements in dietary intake of fruits and vegetables (FVs) and fiber; reductions in total fat or daily fat sources; increases in achieving an anti-inflammatory diet, with Mediterranean diet score; and increased blood carotenoids, indicating greater dietary intake of FVs. In addition, studies showed evidence for reductions in waist circumference and weight loss in the short term (three to six months post-intervention). While studies did not provide evidence for decreasing pro-inflammatory biomarkers, one study showed a borderline effect in global DNA methylation (Cocinar para Su Salud), and another study showed cardiometabolic benefits of greater weight loss and fat loss, with improvements in insulin, glucose, and HOMA-IR at six months and improvements in glucose and an inflammatory marker related to cancer progression at 12 months (e.g., IGF-1 BP1) [40].

A common issue related to limited efficacy in nutrition interventions studies with Latino/as include lack of power to detect effects due to small sample sizes. Two previous reviews included RCT studies with relatively small samples (*n* = 18 or *n* = 20 participants) [22,24], while a different review by Parsons et al. restricted eligibility to interventions with samples sizes greater than or equal to 50 participants [23]. The current review was mostly pilot studies (*n* = 8 of 10 studies). Specifically, there were five studies with 100% Latino/a samples [36,38,44,47,48], and four of the five studies were pilots (*n* = 32 to 80) for samples of 100% Latino/a cancer survivors (Avanzando Juntas [36], Cocinar para Su Salud [38,39,40]), My Health [47], and Nuestra Salud [48]). Three additional pilot studies (BOLD [37], La Activa Vida [41], and Prescription (Rx) for Better Breast Health [49,50]) focused on Black and Latino/a cancer survivors with subgroups of Latino/a cancer survivors between 22 and 79 Latino/a participants. Given this issue of small sample sizes, future studies might consider strategies for recruitment and retention like community-engaged approaches that include survivors as advisors to provide guidance and direction to enhance engagement. Loss to follow-up also may have made it difficult for interventions to show effects.

An important finding was that nutrition interventions for Latino/a cancer survivors in the U.S. have tended to focus on breast cancer survivors. This review identified one study focused on ovarian cancer (LIVES [42,43]), one study (Avanzando Juntas [36]) focused on breast and gynecological cancer, and one study focused on various cancers (75.7% survivors of breast cancer, BOLD [37]). While there has been an overall decline in cancer deaths nationally, important disparities exist [54]. Prior research has shown that the distribution of cancer type is different among Latino/a adults compared to non-Hispanic/Latino/a adults [55]. For example, among non-Hispanic White adult females, the most common cancers are breast, lung, and colorectal, but among Hispanic/Latino/a adult females, the most common cancers are breast, colorectal, and lung [55]. In non-Hispanic White males, the most common cancers are prostate, lung, and colorectal, but in Hispanic adult males, colon cancer replaces lung cancer as the second most common cancer [55]. Regarding mortality, breast cancer is the leading cause of cancer-related deaths among Hispanic females compared to non-Hispanic White females and the second leading cause of death [55]. These data show that incidence rates are lower among Latinas; however, this population is at greater risk of dying from breast cancer [55]. Due to the significant disparities in cancer outcomes, in addition to differences in the distribution of common cancer types among Latino/a adults, it is important to highlight incidence and mortality cancer rates in less common cancer types [55]. For example, while liver cancer is not the most common cancer, Latino/a adults have a two-fold increase in the incidence of liver cancer and mortality compared to non-Hispanic White adults. Furthermore, there is a higher incidence of uterine cervical, kidney, and stomach cancers which corresponds to increased mortality rates among Hispanic adult males and females, apart from kidney cancer [55]. There is a need for nutrition interventions for Latino/a cancer survivors to go beyond breast cancer and consider how to support survivors of cervical, liver, or stomach cancer.

Another important observation was that there were no interventions for rural communities and relatively few nutrition interventions conducted in the Southern, Western, or Southwestern regions of the U.S., including states of California and Texas, which have proportionally more residents of Latino/a heritage [2]. Specifically, there were no nutrition interventions identified in this review, conducted in California, despite having the largest number of National Cancer Institute (NCI)-funded cancer centers in the country (ten total cancer centers and seven comprehensive cancer centers) [56], and despite the lead author asking subject matter experts to help identify relevant interventions for Latino/a cancer survivors in California. There was only one intervention conducted in Texas conducted by Ramirez and colleagues [49].

Among the 10 nutrition interventions, studies primarily relied on subjective, self-reported data for dietary assessment. Some studies used standardized dietary assessment tools like dietary screeners and FFQ, while others used tools created or validated for Latino/a populations. In My Health [47], the research team used the 23-item Brief Dietary Assessment Tool for Hispanics, which was developed specifically for this population. Greenlee and colleagues measured dietary intake with the Block Questionnaire [41], used in the National Health and Nutrition Examination Survey (NHANES), which includes foods common in diets of U.S. Latino/a adults. Other studies did not describe the extent that standardized dietary assessments captured important foods or preparations commonly used by Latino/a adults.

Importantly, there was limited information on biomarkers. This review found four studies that included a biomarker of dietary intake of FVs, carotenoid concentration from blood samples; and six studies included biomarkers related to cardiometabolic health and inflammation. Biomarkers provide important insights regarding bio-behavioral pathways for cancer progression, but of the seven studies that collected biomarkers related to cardiometabolic health and inflammation, only two studies have reported findings for intervention effects on biomarkers [40,52]. To improve health outcomes, future research is warranted to investigate and share the effects of nutrition and physical activity interventions on cancer-related biomarkers among Latino/a cancer survivors [50].

There have been three recent and relevant nutrition reviews for Latino/a cancer survivors [22,23,24]. Pichardo et al. led a scoping review of diet and physical activity interventions, with randomized study designs only, for Black and Latina women after a diagnosis of breast cancer and included studies through October 2022 [24]. Parsons et al. completed a systematic review of nutrition and dietary interventions for the prevention and treatment of cancer, including dietary supplements, nutrition support (including oral nutrition supplements), and nutrition interventions from 2000 to August 2022 [23]. Importantly, their review was not intended to provide evidence for cancer survivorship and excluded interventions that started after cancer treatment. Moreover, their review, while comprehensive, did not focus on minority cancer survivors generally or Latino/a cancer survivors specifically [23]. Castro-Edgin and Agudo conducted a systematic review and meta-analysis of interventions to understand the role of diet for cancer prognosis among adults living in the U.S. and around the world; they searched for observational studies and trials conducted between January 2011 and August 2021; their review was not focused on Latino/a cancer survivors [22]. Samuel and colleagues conducted a systematic literature review of quality of life among Latino/a cancer survivors and called attention to psychosocial and sociocultural factors influencing quality of life, including discrimination, acculturation, and social support, as key factors for quality of life among Latino/a adults living with cancer [57]. Across the three review papers [22,23,24], the included interventions for Latino/a cancer survivors were feasibility or pilot interventions. Growing attention is warranted given the health disparities that exist for Latino survivors, and there have been limited efficacy or effectiveness interventions.

In the early 1990s, Ramirez and colleagues conducted Cuidando Su Corazon, one of the first RCTs to engage Latina women in health promotion [58,59]. This culturally relevant and family-based behavioral nutrition intervention engaged 168 Latina participants for weight loss [58,59]. Scholars had designed, implemented, and evaluated nutrition interventions for cancer prevention with Latina/o communities, such as Mujeres Felices por Ser Saludables [60] and Nuestra Cocina: Mesa Buena, Vida Sana (Our Kitchen: Good Table, Healthy Life), which was conducted along the U.S.–Mexico border [61]. Approximately twenty years later, Paxton and colleagues published one of the first studies on the effects of a nutrition intervention for minority cancer survivors participating in WHEL [51], but to date, there have been relatively few nutrition interventions designed specifically for Latino/a adults for cancer survivorship.

This review was based mostly on pilot studies (*n* = 8 of the 10 studies). Specifically, this review identified five trials with 100% Latino/a cancer survivors (Avanzando Juntas [36], Cocinar para Su Salud [38,39,40], Mi Vida Saludable [44,45,46], My Health [47], and Nuestra Salud [48]), and four of these five were pilots. Three additional pilot trials focused on Black or Latino/a cancer survivors (BOLD [37], La Vida Activa [41], and Prescription (Rx) for Better Health [49,50]). Ongoing trials obtained from ClinicalTrials.gov [30], including a trial with the Vida Plena study [62,63,64]; the COACH [65], the ¡Vida! Program with SMART design [66]; and TEAL [67] interventions, were not included in this review (See Supplementary Table S3), but their findings will contribute critical evidence for Latino/a cancer survivors in the coming years.

### 4.1. Key Messages

Based on this scoping review, there are three key messages to advance nutrition intervention and implementation science for Latino/a cancer survivors in the U.S. specifically and minority cancer survivors more generally. Webb Hooper and Pérez-Stable, leaders of the National Institute on Minority Health and Health Disparities (NIMHD), outlined five strategies for research and practice to advance health equity: (1) increase workforce diversity, equity, and inclusion (DEI); (2) prioritize inclusive research participation; (3) increase cultural competence and humility; (4) apply community-engaged research principles; and (5) go beyond “do no harm” [68]. First, diverse teams are needed to design, implement, and evaluate nutrition interventions for Latino/a cancer survivors for innovation and to help mitigate unintended consequences [68]. Future interventions would benefit from diverse teams, comprising investigators, research assistants, and community partners with shared sociocultural identities as the characteristics of the target population of the intervention, including promotora/es de salud and Latino/a community health workers, and, ideally, inviting team members with lived experiences of cancer. There are examples of community-engaged and culturally relevant behavioral nutrition interventions for Latino communities that collaborated with promotora/es [69,70]. Given the disparities in NIH funding by gender, racial, and ethnic identity [71], policy changes and additional funding may be instrumental in training/mentoring early-career scientists from underrepresented backgrounds and funding diverse teams to lead this important research.

Second, there is a need for community-engaged and culturally relevant interventions that prioritize community engagement and the inclusion of Latino/a cancer survivors and share findings about the effects [68]. Half of the studies described explicit community engagement to create culturally appropriate or culturally tailored interventions (Avanzando Juntas [36]; BOLD [37]; My Health [47]; and the efficacy and effectiveness trials, Cocinar para Su Salud and Mi Vida Saludable, respectively [44]). More diverse teams of investigators, research assistants, and community partners will help with diversity and inclusion in clinical trials, but special attention is warranted to ensure equitable representation in clinical trials for cancer [72,73]. In addition to the strategies outlined by Webb Hooper and Pérez-Stable from the NIMHD [68], the imperative for equitable representation in clinical trials aligns with the broader call to enhance diversity, inclusivity, and community engagement, ultimately advancing health equity for Latino/a cancer survivors and minority populations [72,73]. Interdisciplinary teams of researchers are also needed to analyze and report on the intervention effects on biomarkers, specifically, to advance the science while supporting transparency and trustworthiness with community members.

In regions with large Latino populations and academic institutions with relevant expertise and capacity, intentional efforts are needed to ensure that the burdens and benefits of research are shared for Latino and non-Latino residents. New policies from funding agencies may be required to shift from intentions to recruit locally representative samples of participants from different racial/ethnic backgrounds. Strategies to support the recruitment, retention, and engagement of Latino/a cancer survivors include community engagement and collaboration with community partners, such as promotora/es [70,74,75]. In this review, there were no interventions that described involvement with promotora/es de salud or Latino/a community health workers. Future research may consider models for engaging with promotora/es in the co-creation of nutrition interventions with Latino/a cancer survivors.

In this review, only some studies were culturally adapted or tailored for Latino/a cancer survivors or considered acculturation (Avanzando Juntas [36]; Cocinar para Su Salud [38,39,40] and its effectiveness trial, Mi Vida Saludable [44,45,46]; and My Health [47]). New interventions would benefit from considering sociocultural factors and culturally relevant approaches in intervention design, implementation, and evaluation, including potentially new measures of dietary assessment developed for Latino/a populations or population subgroups. Generally, studies in this review used standardized dietary assessment with existing diet surveys or FFQs. New qualitative or mixed methods studies may explore specific experiences of Latino/a cancer survivors in aligning dietary intake with recommended dietary patterns for survival or investigate resources and constraints related to achieving and maintaining the nutritional targets outlined in ACS or AICR guidelines. For example, Nuestra Salud assessed food insecurity [48], which is an important factor for nutrition in the context of cancer care [76]. Future research can provide insights into whether and how recommendations for cancer survivorship are relevant for Latino/a cancer survivors, which is supported by others emphasize the need to consider underlying health determinants (e.g., system, structural, and social determinants of cancer) [4].

Third, mobile (“mhealth”) and ehealth applications offer immense promise for improving care and outcomes among cancer survivors [77,78] by alleviating barriers to recruitment, retention, and engagement in nutrition interventions with Latino/a cancer survivors. However, few studies have leveraged this approach with Latino/a cancer survivors. In this review, only one study that used m/ehealth to deliver a nutrition intervention among Latino/c cancer survivors (the My Health app) [47] and another study, Mi Vida Saludable, applied an ehealth approach testing text messages and newsletters linked to the project’s website in addition to in-person group sessions [44,45,46]. This review found two other m/ehealth interventions on ClinicalTrials.gov [30], but one trial was terminated, and another had not started recruitment. The Mi Saludable en Mi Valle intervention was an ehealth nutrition and physical activity intervention for rural Latino cancer survivors in Washington State; however, per ClinicalTrials.gov, this trial stopped due to challenges with recruitment [79]. The ¡Vida! Program is another m/ehealth intervention; it is specifically a tailored weight loss intervention for Latina breast cancer survivors, but the study had not started recruiting at the time of this review [66]. Opportunities to explore m/ehealth interventions among Latino/a cancer survivors remain untapped.

### 4.2. Limitations and Strengths

This review had some limitations. The findings from this review were based on a total of 748 Latino/a participants from 10 studies and cannot be generalized to all communities or populations of Latino/a cancer survivors in the U.S. or outside the U.S. There was a trade-off between an inclusive review that reflected more of the available evidence and a more restricted review that included more homogenous interventions and potentially higher-quality evidence from larger or more rigorously designed interventions, especially RCTs. The variability in study designs and outcome measures across the included studies presented challenges in synthesizing evidence. This review was not able to assess threats to validity, such as non-compliance, for included studies. The search process, while comprehensive, might have missed some relevant studies. This review excluded two studies, because the total number of Latino/a cancer survivors in the subgroup was less than 20 participants. For example, the ALIVE study, an eHealth trial primarily for African American breast cancer survivors with separate tracks for nutrition and PA, reported engaging 12 cancer survivors of Hispanic and mixed race/ethnicity (11% of total sample) [80]. The MEAL study, a trial testing a high-vegetable dietary pattern for primarily White prostate cancer survivors, included 17 Hispanic male cancer survivors (4% of total sample) [81,82]. In addition, this review was not able to consider all information from relevant studies, due to the small sample sizes of Latino/a subgroups in studies with mixed samples, or rules about data sharing or publishing findings for recent or ongoing trials. Half of the studies (*n* = 5) were mixed samples of Latino/a and non-Latino/a cancer survivors, but only one, the subgroup analysis of WHEL, reported results specifically for the Latino/a participants [51]. Because the sample size of the Latino/a subgroup was relatively small, there were no additional subgroup analyses for pilot studies: BOLD (*n* = 22) [37], La Vida Activa (*n* = 33) [41], or Prescription (Rx) for Better Breast Health (*n* = 79) [49,50]. The LIVES Study did not have subgroup analyses due to strict limitations on sharing results (*n* = 63) [42,43]. Moreover, there is a balance between rigor in outcome assessment and respondent burden, especially when considering issues of acceptability, logistics, and cost with tissue and blood collection among cancer survivors. While seven studies measured biomarkers as objective measures of outcomes, not all studies reported on the effects. Intervention impacts were limited by available results. Lastly, this review focused on Latino/a cancer survivors in the U.S. Additional research is warranted to understand gaps and opportunities for cancer care for Latino/a cancer survivors living outside the U.S. and other population subgroups experiencing inequalities in cancer care.

This review had several strengths. First, the review was conducted by an interdisciplinary team with expertise in nutrition and cancer, and specifically behavioral change interventions, nutrition, and cancer among Latino/a populations. Second, the scoping review process followed a rigorous methodological framework [26,27,28] and the PRISMA Statement for Scoping Reviews [25]. A comprehensive search of the peer-reviewed literature and a search of the gray literature, including an outreach campaign to reach subject-matter experts, enabled this review to identify more of the relevant interventions. The rigorous process also helped to minimize selection bias and support the validity of findings. Third, this review utilized more detailed data extraction to summarize critical details needed for intervention design, implementation, and evaluation. The research team also consulted with experts to obtain insights beyond data extraction, as recommended [27].

### 4.3. Implications

The implications for this review relate to research, practice, and policy in the U.S. First, future research is needed to design, implement, and evaluate community-engaged, culturally relevant, and theory-informed interventions for Latino/a cancer survivors living in the Southwestern and Southern regions of the U.S., with large proportions of Latino/a residents in places like California and Texas [2], and in other states with growing populations of Latino/a residents, including in the Southern region of the U.S. (e.g., Alabama, South Carolina, and Kentucky) [83]. To date, there have been no published studies of completed trials for Latino/a cancer survivors residing in rural communities. There are unrealized opportunities to increase capacity for and conduct trials in collaboration with cancer centers near rural populations, especially in states in the Southern region, like Florida and Texas, and center community engagement through community advisory boards or collaboration with promotora/es. Future interventions, specifically efficacy and effectiveness trials, are needed to go beyond breast cancer and focus on survivors of cervical, liver, and stomach cancer; intentionally prioritize recruitment and engagement for rural residents; and assess follow-up outcomes both early on and later on in survivorship, beyond five years. For interventions focusing on inflammation, additional consideration is needed to select, collect, and analyze relevant biomarker data and share findings about intervention effects on biomarkers. In addition to behavioral goals related to dietary intake of fruits and vegetables, researchers might consider including goals for other food groups recommended by ACS and AICR, such as beans and lentils [17,19], a rich source of fiber [84], which is particularly relevant for fiber-related cancers among Latino/a adults, such as colorectal cancer.

Second, intervention treatments have been traditional in testing varied forms of nutrition education or experiential nutrition education. Future research might consider examining the effects of nutrition education or behavioral nutrition by using motivational interviewing principles, in combination with other treatments, such as chemotherapy or pharmaceuticals like metformin, to improve cardiometabolic indicators related to inflammation and cancer for Latino/a cancer survivors. For example, the cause of chemotherapy-associated weight gain is multifactorial and may result from changes in fluid retention, physical activity, metabolic dysregulation, and changes in nutrition-related behaviors [85,86]. Weight gain is particularly important for individuals requiring hormone-based therapies for breast or prostate cancer, the most common cancer among men and women, because of its impact on cancer progression and recurrence. Further, metabolic and inflammatory changes associated with chemotherapy and treatment-related weight gain can lead to other long-term health outcomes, including cardiovascular disease, diabetes, and bone loss [85,86]. Previous studies have shown that metformin could be beneficial among breast cancer survivors at high risk for diabetes, and it could have potential heart-protective effects [85,86]. Intervention studies could be designed to investigate the potential benefit of layered interventions, including nutrition and metformin, to target breast cancer survivors who are at high risk of diabetes to determine an optimal benefit. Combining nutrition education or behavioral nutrition with chemotherapy or pharmaceuticals may improve cancer-related outcomes and potentially benefit other long-term health outcomes, including cardiometabolic health and bone health.

Third, researchers might design interventions for different dietary patterns and test effects on cancer-free survival, well-being, or other relevant outcomes. Prior research has shown the positive effects of a plant-based dietary pattern, characterized by a higher intake of fruits and vegetables, with more carotenoids and fiber, on dietary intake of FVs and fiber, but not on cancer recurrence or cancer progression [51,87]. However, the dietary pattern tested was based on nutrition science of the 1990s, and new interventions might consider culturally based dietary patterns. Mattei and colleagues are studying the importance of traditional and cultural foods, sociocultural attitudes and preferences, and culturally relevant dietary assessment for translational research applications [88,89,90], such as developing culture-centered dietary interventions based on cultural humility [91]. Nutrition professionals can play important roles in interpreting standardized nutrition guidelines (e.g., ACS and AICR guidelines) into meaningful recommendations for specific individuals and communities they serve, acknowledging differences in sociocultural values, foods, and behaviors within and across racial/ethnic groups. For example, AICR guidelines emphasize eating a diet rich in beans and lentils, and nutrition professionals can support healthy eating by suggesting relevant options for beans and lentils based on cultural dietary patterns. Future directions may consider how nutrition professionals in research and practice can effectively promote culturally relevant recommendations for healthy survivorship.

Another possibility might be to consider other dietary factors, like protein intake-associated dietary acid load in a dietary pattern for cancer survivorship. Preclinical models have shown that a dysregulated acid–base balance can lead to metabolic and tumor acidosis, a physiological condition associated with cancer progression, with a high diet acid load (DAL) [92,93,94,95]. However, the contribution of diet and tumor acidosis is not fully understood. Briefly, a high DAL would be characterized as a diet high in protein and meat intake and lower in fruits and vegetables. Conversely, a low DAL is characterized by a reduced meat and protein intake with higher fruit and vegetable intake, which is a pattern in alignment with several AICR cancer prevention guidelines [19,96,97]. Importantly, Latino/as have a high DAL compared to non-Hispanic Whites [98]. However, few studies have assessed the role of DAL among cancer survivors. Wu and colleagues have examined dietary acid load and acid-producing diets on inflammation for breast cancer survivors, based on data from the WHEL study [99,100]. Future nutrition interventions might consider exploring to what extent these dietary patterns mitigate inflammation, hormonal, and metabolic perturbations, and adverse cardiometabolic outcomes linked to cancer recurrence among Latino/a cancer survivors. Qualitative or mixed methods studies also may explore specific challenges that Latino/a cancer survivors have in aligning dietary intake with recommended dietary patterns/achieving and maintaining the nutritional targets outlined in cancer-specific nutrition guidelines.

Regarding practice, health providers from medicine, public health, and other disciplines depend on high-quality evidence to make evidence-based decisions for their patients or clients, and ultimately, for the families and communities that support them. Despite a comprehensive search process, there is limited evidence on the potential benefits of nutrition interventions for Latino/a cancer survivors in the U.S. (*n* = 10 included studies in this review), though findings suggest some potential benefits for nutrition and well-being. Practitioners may consider integrating existing key messages around nutrition from ACS and AICR cancer guidelines [17,19] and working with community partners, including community advisory boards, community health workers, and promotora/es, to design, implement, and evaluate interventions, specifically for Latino/a cancer survivors in their local communities or regions [75]. Collaborations between clinical, community, and academic partners can lead to enhanced engagement and cultural appropriateness, inform better science, and support equity [68,74]. Going forward, practitioners can serve as critical partners, advocating for diversity and inclusion priorities with people in power and access to resources.

Policy implications relate to diversity and inclusion in the U.S. research enterprise. Specifically, while NIH policy requires inclusion of minority participants, unless there is a scientific or ethical reason for exclusion [101], there have been relatively few trials for diverse samples of cancer survivors. This review identified only five trials with 100% Latino/a samples, and all were feasibility or pilot studies (Avanzando Juntas [36], Cocinar para Su Salud [38,39,40], Mi Vida Saludable [44,45,46], My Health [47], and Nuestra Salud [48]). Three additional trials focused on Black or Latino/a cancer survivors (BOLD [37], La Vida Activa [41], and Prescription (Rx) for Better Health [49]), and they were also designated as pilot or feasibility studies. While ongoing trials will contribute critical evidence for Latino/a cancer survivors (Supplementary Table S3), large-scale changes are needed in research. A strategic effort is required at all stages to recruit and retain individuals from underrepresented backgrounds; cross-train them in nutrition, intervention, implementation science, science of cancer, and science of health disparities; mentor them in the grant writing and review process; provide adequate support for them to lead impactful interventions for cancer survivorship with underserved communities; and eliminate barriers to their leadership in the field [68]. Moreover, there are more National Cancer Institute (NCI) funded cancer centers in California than any other state [56], and despite a large Latino/a population [102], this review did not find any trials conducted in California. Other large states, with large proportions of Latino/a residents, have fewer NCI-funded cancer centers than California. For example, Florida and Texas each have four NCI-funded cancer centers currently [56], and there were no interventions identified from Florida and only one intervention based in Texas [49]. Changes to policies within funding organizations and university research operations will help reduce barriers to hiring, paying, and valuing community members (e.g., promotora/es) and facilitate community-engaged research. Future policies likely need to incentivize community engagement and representative samples of intervention participants, especially for states in the U.S. with large Latino/a populations, to accelerate the science of cancer disparities and cancer equity.

## Figures and Tables

**Table 1:** Nutrition interventions with Latino/a cancer survivors: sample and setting of included studies.

	Authors (Year)	Name of Intervention (Study Dates; NCT Number)	Focus	% Subgroup in Total Sample (*n*); Total Sample (*n*)	% Female	% Cancer Type	Cancer Stage and Active Treatment	Location	Intervention Site
1	Stolley (2020) [36]	**Avanzando Juntas/Moving Forward** (2019–2022) ^a^ NCT04321135 ^b^	Culturally adapted and community-based weight loss (nutrition and PA)	100% Hispanic or Latina survivors (*n* = 32) ^c^	100% female	100% breast cancer and Gynecological cancer	Stage 0–III breast and gynecological cancer; at least 3 months post-treatment	Milwaukee, WI	Community site
2	Conlon et al. (2015) [37]	**Bronx Oncology Living Daily (BOLD) Healthy Living** (2011–2012) No NCT	Community-based diabetes prevention and control (nutrition and PA)	26.5% Hispanic/Latino survivors and co-survivors (*n* = 22); total sample (*n* = 83) survivors and co-survivors	95.2% female	75.7% breast cancer; 6.1% gynecological cancer; 6.1% lung; 30.1% Other cancer type	No additional criteria	Bronx Co., NY	Community site
3	Greenlee et al. (2015) [38,39,40]	**Cocinar para Su Salud (Cook for Your Health/Life)** (2011–2012) NCT01414062 ^b^	Culturally based and community-based lifestyle (nutrition only)	100% Hispanic survivors (*n* = 70) All Spanish speakers	100% female	100% breast cancer	Stage 0–III breast cancer; at least 3 months post-treatment	New York City, NY	University site (e.g., teaching facility at Columbia University)
4	Greenlee et al. (2013) [41]	**La Vida Activa/An Active Life** (2011–2012) NCT00811824 ^b^	Weight loss (nutrition and PA)	79% Hispanic survivors (*n* = 33); total sample (*n* = 42)	100% female	100% breast cancer	Stage 0–IIIa breast cancer; at least 6 months post-treatment	New York City, NY	Community site ^d^
5	Thomson et al. (2023) [42,43]	**LIVES (Lifestyle Intervention for Ovarian Cancer Enhanced Survival)** (2012–2018) NCT00719303 ^b^	Cancer-free progression (nutrition and PA)	5.5% Hispanic survivors (*n* = 63); total sample (*n* = 1205)	100% female	100% ovarian cancer	Complete clinical remission of ovarian cancer (stage II–IV); Gynecological Oncology Group Performance Grade of 0–2; between 6 weeks and 6.5 months post-treatment	Multisite; recruited participants from NRG sites across the U.S. and Canada	Remote only by telephone and internet (with electronic health and intervention platform, eHIP)
6	Greenlee et al. (2022) [44,45,46]	**Mi Vida Saludable/My Healthy Life** (2016–2020) NCT02780271 ^b^	Culturally tailored and community-based lifestyle (nutrition and PA)	100% Hispanic or Latina survivors (*n* = 167)	100% female	100% breast cancer	Stage 0–III breast cancer; at least 3 months post-treatment	Columbia University Medical Center New York, NY	Hybrid; clinical site (e.g., Columbia University Medical Center (CUMC)) and remote by phone/internet (with electronic health and intervention platform, eHIP)
7	Yanez, et al. (2020) [47]	**My Health** (2015–2019) NCT03645005 ^b^	Culturally based and community-based lifestyle (nutrition and PA)	100% Latina survivors (*n* = 80)	100% female	100% breast cancer	Stage 0–III breast cancer; within 2–24 months of completing treatment.	Chicago, IL	Remote only by app/internet
8	Crane et al. (2020) [48]	**Nuestra Salud/For Your Health** (2018–2019) ^a^ NCT04314479 ^b^	Lifestyle and symptom management (nutrition and PA)	100% Latina survivors (*n* = 37) and caregivers without cancer; total sample (*n* = 71)	100% female	81% breast cancer (*n* = 30) Included cancer survivors of head or neck, liver, colon, kidney, and lymphoma	No eligibility related to cancer stage. Completed primary treatment for solid tumor cancers and included participants undergoing active treatment (*n* = 6 cancer survivors)	U.S.–Mexico border Southern AZ	Remote only by telephone and internet (with electronic health and intervention platform, eHIP)
9	Ramirez et al. (2017 [49]); Zuniga et al. (2019) [50]	**Prescription (Rx) for Better Breast Health** (2013–2017) ^a^ NCT02279303 ^b^	Patient-navigated, anti-inflammatory, culinary-based (nutrition only)	51.2% Hispanic survivors (*n* = 79); total sample (*n* = 153) All English speakers	100% female	100% breast cancer	Early stage (0–III); At least 2 months post-treatment	San Antonio, TX	University site (e.g., Institute for Health Promotion Research at UT Health San Antonio) and remote by telephone
10	Pierce et al. (2002) [51]; Paxton et al. (2011) [52]	**Women’s Health Eating and Living Study (WHEL)** (1995–2000) No NCT ^b^	Cancer-free progression (nutrition only)	5.3% Hispanic survivors (*n* = 165); total sample (*n* = 3088) All English speakers	100% female	100% breast cancer	Stage I–IIIA breast cancer within 4 years of study entry. Completed treatment with no evidence of recurrent disease	Multisite. Recruited participants from CA, AZ, OR, and TX	Community/clinical site (details NR) and remote by telephone

Research assistants extracted information for studies from published abstracts and journal articles, except when indicated by footnotes. Study dates are for the enrollment or intervention period described in the original research record or available from ClinicalTrials.gov or Reporter.NIH.gov websites accessed on 28 September 2023. Regarding sample size, the number is the number enrolled or randomly assigned to intervention groups. Labels for race/ethnicity are consistent with the original research and different studies reported race/ethnicity differently. Location is geographic region where trial was conducted. Intervention setting is site of intervention, such as clinical, community, or remote via telephone or internet. ^a^ Information obtained from entry in ClinicalTrials.gov. ^b^ Information obtained from NIH RePORTER tool Reporter.NIH.gov. ^c^ Information obtained from personal communication. ^d^ Described as a “community situated intervention” and categorized as community. AZ, Arizona; CA, California; eHIP, eHealth and intervention platform; IL, Illinois; NCT, ClinicalTrials.gov identifier; NR, not reported; NY, New York; OR, Oregon; PA, physical activity; RCT, randomized controlled trial; TX, Texas; WI, Wisconsin.

**Table 2:** Nutrition interventions with Latino/a cancer survivors: theory, delivery, and dose.

	Intervention Name	Theory	Intervention Components	Delivery	Interventionist	Primary Outcome	Secondary Outcome for Well-Being and Quality of Life (QOL)	Estimated Dose Delivered
1	**Avanzando Juntas**/**Moving Forward** [36]	NR ACS guidelines for nutrition and PA included in informational binder.	Culturally adapted and community-based nutrition education class with weekly text messages for reinforcement. ^a^	F2F (group classes) and remote/virtual via text messaging (individual). ^a^ Language: bilingual. ^a^	NR	Dietary Intake: fruits and vegetables, red meat, and processed meat; overall dietary quality assessed with HEI (self-administered 24 h dietary recall with ASA24). ^a^	HRQOL (Patient-Reported Outcomes Measurement Information System, PROMIS)	180 min (3 h) sessions × 16 sessions over 16 weeks = 2880 min (48 h). Note: Weekly text messages not counted in dose (2–3 per week) ^a^ Dose: 48 h
2	**Bronx Oncology Living Daily (BOLD) Healthy Living** [37]	Social–ecological framework RE-AIM Framework ^b^ ADA, ACS, and AICR guidelines used in curriculum	Community-based nutrition education class and group exercise.	F2F group class. Language: bilingual.	Health-care professionals, including RDNs, EP, and certified trainers.	Body composition: Height, weight, WC (direct observation).	HRQOL (2-items from the SF-36), including perceived health status and perceived pain.	Dose: NR
3	**Cocinar Para Su Salud/Cook for Your Health/Life** [38,39,40]	DESIGN Framework. ^c^ Transtheoretical Model (TTM, stages-of-change construct). Social Cognitive Theory (SCT). AICR and ACS guidelines used for curriculum.	Culturally tailored nutrition education class, cooking class, and food shopping field trips with monthly telephone calls from study coordinator (RD) for retention.	F2F group class. Language: Spanish only.	A bilingual, Latino/a RD and bilingual Latino/a chef.	Dietary intake: Fruits and vegetables (24-h dietary recalls) and percentage of energy from total fat; Biomarker of dietary intake of FV (blood carotenoid concentration).	None	1.5-to-3.5 h sessions × 9 sessions (24 h total for session) over 12 weeks Note: Telephone calls not counted in dose. Author-reported total dose. Dose: 24 h
4	**La Vida Activa/An Active Life** [41]	NR	Curves Weight Management Program with use of Curves fitness centers and a Curves diet plan, with Curve curriculum with books, DVDs, and instructor’s manual plus nutrition courses for study participants and weekly motivational telephone calls from instructor.	F2F group class. Language: bilingual.	Nutrition courses led by bilingual instructor and exercise classes led by Curves staff and trainers.	Body composition: weight change (weight loss).	None	1 h nutrition sessions × 6 sessions over 6 weeks and additional contacts for exercise. Note: Exercise classes and telephone calls not counted in dose because number and duration varied. Dose: 6 h
5	**LIVES (Lifestyle Intervention for Ovarian Cancer Enhanced Survival)** [42,43]	SCT. Theory of Planned Behavior. ACS guidelines used for nutrition goals and AICR and ACS guidelines for PA goals.	Tele-coaching, based on MI, with text messages and emails for reinforcement.	Remote/virtual via smartphone (calls and texts) and internet-based (email). Language: bilingual.	Health coaches (undergraduate students majoring in nutrition science or dietetics and trained in evidence and behavior theory-based strategies, including MI, for 6 weeks)	Progression-free survival or death from any cause (measured: the number of months between study enrollment and documentation of disease progression).	QOL (RAND-36)	33 coaching sessions × NR minutes/session over 24 months with a phased approach. Note: Text messages not counted in dose. Dose: NR
6	**Mi Vida Saludable**/**My Healthy Life** [44,45,46]	DESIGN Framework. ^c^ TTM. SCT. AICR and ACS guidelines used for curriculum.	Culturally tailored nutrition education classes, hands-on skills-building exercises, field trips, written materials about ACS and AICR guidelines, FitBit for self-monitoring of PA, text messages, emails, access to interactive project website, with monthly telephone calls for retention.	F2F or remote/virtual via smartphone (texts) and internet. The ehealth components included 2–3 text messages per week and 2 e-newsletters per month sent via eHIP and linked to the project’s website for 11 months. Language: bilingual.	Intervention staff include a trained chef, a nutrition and physical activity educator, and a dance-class instructor.	Dietary intake: intake of FVs and total energy density (interviewer-administered 24-h dietary recalls). Biomarker of dietary intake of FV (blood carotenoid concentration).	PROMIS Scale v1.2—global health and PROMIS-43 profile v2 for QOL, including physical, mental, and social health.	Note: Study included different arms with varied contacts. Minimum dose for F2F: 16 h in the first month. Minimum dose for ehealth: NR. Dose: NR
7	**My Health** [47]	Quality-of-Life Cancer Survivorship Framework.	My Health app plus tele-coaching calls with tele-coach trained in MI (3 calls before weeks 1, 2, and 6, and for low-app-usage participants, additional calls at weeks 3, 4, and 5). Culturally appropriate materials.	Remote/virtual via app on smartphone or internet platform. Language: bilingual.	Bilingual health coaches trained by a licensed clinical psychologist in MI, problem solving, and goal setting.	Dietary intake: intake of fruits and vegetables and fewer daily fat sources (23-Item Brief Dietary Assessment Tool for Hispanics).	None	2 h of app use per week over 6 weeks = 12 h Note: Three to six tele-coaching calls not counted in dose. Dose: 12 h
8	**Nuestra Salud/For Your Health** [48]	SCT and integrated symptom management and lifestyle intervention (SMLI). ACS guidelines used for nutrition and PA goals.	Telephone coaching sessions, based on MI, (each session 20–30 min) and text messages delivered via eHIP and FitBit for self-monitoring of PA.	Remote/virtual via telephone calls and text messages. Language: bilingual.	Bilingual and bicultural health coaches (≥4 years of experience)	Dietary intake: Usual diet over past 30 days (19-items from NCI DSQ). PA: frequency and duration over the last 7 days (9-items from the WHI PAQ).	None. (Study used PROMIS to assess self-efficacy for symptom management.)	12 coaching sessions × 25 min/session over 12 weeks = 300 min Dose: 5 h
9	**Prescription (Rx) for Better Breast Health** [49,50]	SCT and Bandura stages of change construct. USDA dietary intake guidelines for five major food groups used for nutrition goals.	Nutrition and cooking anti-inflammation workshops (“dietary workshops), MI (individually tailored) tele-coaching sessions, and tailored newsletters based on stages of change for reinforcements.	F2F (group classes) and remote/virtual via tele-counseling (individual). ^a^ Language: English only.	Workshop facilitator: NR. A chef skilled in anti-inflammatory food preparation led culinary cooking demonstrations. Coaches were patient navigators and trained in MI by certified member of the MI Network of Trainers.	Inflammation: CRP and IL-10.	Physical, social/family, emotional, and functional well-being (subscales from the Breast Cancer Functional Assessment of Cancer Therapy Scale (FACT-B).	6-monthly workshops × NR minutes/workshop + monthly MI coaching sessions × minutes/session within 4 weeks after each workshop for first 6 months and every month over 12 months Dose: NR
10	**Women’s Healthy Eating and Living Study (WHEL)** [51,52]	SCT	Cooking classes, tele-counseling, and monthly newsletters.	F2F (Group classes) and Remote/virtual via tele-counseling (individual) ^a^ Language: English only.	Counselors (for tele-counseling) trained in MI.	Cancer recurrence, new primary breast cancer, and death from any cause (interviewer-administered survey and confirmation of recurrence using medical records).	QOL (RAND-36 scale included in the Thoughts and Feelings Questionnaire).	Note: A stepped intervention with three phases. The number of tele-counseling sessions varied. Dose: NR

Research assistants extracted information for studies. Theory, model, or framework was reported by authors for the intervention design, implementation, or evaluation. Intervention components included intervention activities to achieve outcomes. Delivery was defined by format for physically in-person or face-to-face (F2F) versus remote or virtually delivered interventions. Hybrid interventions were marked with a footnote. Interventions included group leaders and counselors leading classes or sessions, including tele-counseling sessions. Primary and secondary outcomes were defined by study authors. Only secondary outcomes related to well-being and QOL; body composition, including weight; dietary intake; and PA are shown in this table. Given the focus of the review on positive health outcomes, such as well-being and QOL, these outcomes are shown separately. The Estimated dose was calculated based on duration of session and number of sessions over the active intervention period. ADA, American Diabetes Association; ACS, American Cancer Society; AICR, American Institute of Cancer Research; Automated Self-Administered 24-h (ASA24^®^) Dietary Assessment Tool; BMI, body mass index; ASA24 DESIGN: Nutrition Education DESIGN procedure based on the steps decide behavior, explore determinants, select theory based model, indicate objectives, generate plans, and nail down evaluation; eHIP, electronic health and intervention platform; EPs, exercise physiologists; F2F, face-to-face; FFQ, Food Frequency Questionnaire; FVs, fruits and vegetables; HC, hip circumference; HEI, Healthy Eating Index; HRQOL, health-related quality of life; MI, motivational interviewing; NE, nutrition education; NHANES, National Health and Nutrition examination Survey; NR, not reported; NS, non-statistically significant; PA, physical activity; RCT, randomized controlled trial; RAND-36, survey instrument developed by RAND Corporation to measure HRQOL; RE-AIM, Reach, Efficacy, Adoption, Implementation, and Maintenance; SF-36: Short-Form Health Survey; SMLI, symptom management and lifestyle intervention; SMSH, Symptom Management and Survivorship Handbook; WC, waist circumference; WHI, Women’s Health Initiative (WHI) Physical Activity Questionnaire. ^a^ Information obtained from entry in ClinicalTrials.gov. ^b^ Information obtained from NIH RePORTER tool Reporter.NIH.gov. ^c^ Framework used for intervention development [44].

**Table 3:** Nutrition interventions for Latino/a cancer survivors: outcomes and effects.

	Intervention Name	Study design (Duration and Timing of Assessment)	Primary Outcome	Secondary Outcome for Well-Being and Quality of Life (QOL)	Additional Secondary Outcomes for Weight or Physical Activity (PA)	Main Effects	Secondary Effects
1	**Avanzando Juntas/Moving Forward** [36]	Pilot RCT (4 months) Baseline, post-test (4 months) No follow-up.	Dietary intake: fruits and vegetables, red meat and processed meat, and overall dietary quality assessed with HEI (self-administered 24-h dietary recall with ASA24). ^a^	HRQOL (Patient-Reported Outcomes Measurement Information System, PROMIS).	Body composition: ratio of lean mass to fat mass (direct observation: measured by Bio-electrical Impedance Analysis Scale). PA: moderate-to-vigorous PA (accelerometry via ActiGraph monitors) and change in resistance training (measured: 30 s Chair Stand).	No: Effects on dietary quality, measured with HEI total or HEI component scores, though within-group increases for total vegetables, greens and beans, whole fruit, total protein, and sodium for intervention group; between-group differences favored total vegetables, greens and beans, and total protein. NS for between-group differences. ^b^	NR ^c^
2	**Bronx Oncology Living Daily (BOLD) Healthy Living** [37]	Pilot RCT (4 weeks–12 weeks). Baseline and 4 and 12 weeks. No follow-up.	Body composition: height, weight, and WC (direct observation).	HRQOL (2 items from the SF-36), including perceived health status and perceived pain.	None	Yes: At 12 weeks, no effects on BMI, but effects for decrease in WC for participants in 12-week vs. 4-week program (41.8 vs. 40.8 inches, *p* = 0.03). ^d^	Yes: At 4 weeks and 12 weeks, effects on perceived health status (*p* = 0.001) and borderline effects on perceived pain (*p* = 0.05). ^d^
3	**Cocinar Para Su Salud/Cook for Your Health/Life** [38,39,40]	Pilot RCT (12 weeks). Baseline and 3, 6, and 12 months. Included follow-up at 6 and 12 months post-baseline.	Dietary intake: fruits and vegetables (24-h dietary recalls) and percentage of energy from total fat.	None	Body composition: height, weight, WC, and HC (direct observation).	Yes: At 3 months, effect on all FVs (+1.1 vs. −0.3, *p* = 0.05) and targeted FVs (*p* = 0.004) for intervention vs. control; effect on % calories from total fat (−7.1% vs. −1.6%, *p* = 0.01) for intervention vs. control. At 6 months, effect on all FVs and targeted FVs maintained; effect on % calories from total fat: NS.	Yes. At 6 months, effect on WC between groups (−1.6 cm vs. +1.7 cm, *p* = 0.05). Effect on weight outcomes: NS.
4	**La Vida Activa/An Active Life** [41]	Pilot RCT (6 months). Baseline and every 3 months through 12 months. Included follow-up at 12 months.	Body composition: weight change (weight loss).	None	Body composition: DEXA, height, weight, HC, and WC. PA: survey for type, frequency, duration, and intensity (Kaiser Physical Activity Survey) and cardiopulmonary exercise stress test (VO2max). Dietary intake (110-item Block questionnaire used in NHANES with food items for Hispanic populations).	Yes: At month 6, women in the intervention group lost an average 3.3% (±3.5%) of body weight (range: 1.7% gain to 10.6% loss), as compared with 1.8% (±2.9%) weight loss in the control group (*p* = 0.04). At month 12, on average women in the IA regained some but not all of the weight lost during the first 6 months (*p* = 0.02). ^d^	Yes: At 6 months, WC decreased, marginally statistically significant (*p* = 0.07). ^d^
5	**LIVES: Lifestyle Intervention for Ovarian Cancer Enhanced Survival** [42,43]	Pilot RCT (6 months). Baseline and every 3 months from baseline through 24 months (2 years). Included follow-up at 24 months.	Progression-free survival or death from any cause (measured: the number of months between study enrollment and documentation of disease progression).	QOL (measured: the RAND-36 ^e^ scale with 36 items and bowel health (Gastrointestinal Symptom Scale).	Dietary intake: usual diet (Arizona FFQ). Body composition: BMI (direct observation). PA: moderate-to-vigorous PA (MET-h/week), sedentary time (h/week), and steps/day (accelerometry and self-reported in Arizona PAQ).	NR ^c,d^	NR ^c,d^
6	**Mi Vida Saludable/My Healthy Life** [44,45,46]	RCT (12 months) Baseline and 6 and 12 months. No follow-up.	Dietary intake: intake of FVs and total energy density (interviewer-administered 24-h dietary recalls). Biomarker of dietary intake of FVs (blood carotenoid concentration).	PROMIS Scale v1.2—global health and PROMIS-43 profile v2 for QOL, including physical, mental, and social health.	PA: moderate-to-vigorous PA (FitBit) and survey, 7-day Physical Activity Recall (7DPAR). Body composition: height, weight, WC, and HC (direct observation).	NR ^c,f^	NR ^c,f^
7	**My Health** [47]	Pilot RCT (6 weeks) Baseline and 6 weeks. Included follow-up two weeks after immediate post-test.	Dietary intake: intake of fruits and vegetables and fewer daily fat sources (23-item Brief Dietary Assessment Tool for Hispanics).	None	PA: moderate-to-vigorous PA (FitBit) and survey, 7-item International Physical Activity Questionnaire Short Form (IPAQ-SF).	Yes: At 6 weeks and 2 weeks later, significant interaction of time and condition on daily fat sources in intervention (My Health) relative to control (My Guide) with *p* = 0.015 and = 0.009, respectively. Average daily fat sources did not significantly differ between groups. No effects on daily servings of FVs.	None
8	**Nuestra Salud/For Your Health** [48]	Pilot RCT (12 weeks) Baseline and 12 weeks No follow-up.	Dietary intake: Usual diet over past 30 days (19-items from NCI DSQ). PA: frequency and duration over the last 7 days (9-items from the WHI PAQ).	None. (Study used PROMIS to assess self-efficacy for symptom management.)	None. (PA was primary outcome.)	Yes: At post-intervention, cancer survivors had medium-to-large effects for goals related to FV servings (*d* = 0.55), vegetables (*d* = 0.72), sugar intake (*d* = 0.51); medium effects (clinically significant) for increases in total minutes of PA per week (*d* = 0.42) and grams of fiber intake (*d =* 0.40).	Yes: At post-intervention, cancer survivors had medium-to-large effects for improved symptom severity (*d* = 0.74).
9	**Prescription (Rx) for Better Breast Health** [49,50]	Pilot RCT (6 months) Baseline and 6 and 12 months Included follow-up at 12 months (1 year).	Inflammation: CRP and IL-10.	Physical, social/family, emotional, and functional well-being (subscales from the Breast Cancer Functional Assessment of Cancer Therapy Scale (FACT-B).	Body composition: body mass index. Dietary intake: Mediterranean Diet Score (14-item from Diet Assessment Tool), total energy intake, % calories from macronutrients, and FV intake. PA: monitoring of PA and inactivity (measured via 14-item IPAQ Short Form, the International PA Questionnaire).	NR ^c,d^	Yes: At 6 months: effects on secondary outcomes change in Mediterranean diet score, +1.6 change in Mediterranean diet score in intervention group vs. +0.2 change in control group (*p* < 0.001). Effects on total energy intake (−195.5 vs. + 34.8, *p* = 0.045). No effects on other dietary outcomes, including FVs and fiber. ^d^
10	**Women’s Health Eating and Living (WHEL) Study** [51,52]	RCT (12 months) Baseline and 12 months Included follow-up annually at year 2, 3, 4, and 6.	Cancer recurrence, new primary breast cancer, and death from any cause (interviewer-administered survey and confirmation of recurrence using medical records).	QOL (RAND-36 QOL scale included in the Thoughts and Feelings Questionnaire).	Body composition: height, weight, HC, and WC (direct observation). Dietary intake: usual diet over the past 3 months (Arizona FFQ). Intake over the past 3 weeks (3 × 24-h dietary recalls for different subsamples). Biomarker of dietary intake of FVs (blood plasma carotenoid concentration) for subsample.	No: At final time point year (average 7.3 years), no effects on cancer recurrence or mortality by race/ethnicity for Hispanic versus White survivors.	Yes: At 1 year and 4 years: effects on secondary outcomes for dietary intake of fiber, FV, and percent energy from fat for Hispanic survivors. Effects on QOL or body composition or weight outcomes: NR ^c^

Research assistants extracted information for studies. There were five author-defined pilot studies and three other studies designated as pilots. Primary and secondary outcomes were defined by study authors. Only secondary outcomes related to well-being and QOL; body composition, including weight; dietary intake; and PA are shown in this table. Given the focus of the review on positive health outcomes, such as well-being and QOL, these outcomes are shown separately. The text on the results summarizes additional secondary outcomes assessed in the ten included studies. Main effects and secondary effects were defined based on a statistically significant effect in the hypothesized direction for the primary and secondary outcomes, respectively; *p*-values for statistical significance varied by study. ASA24, Automated Self-Administered 24-h (ASA24^®^) Dietary Assessment Tool; CRP, C-reactive protein, a pro-inflammatory biomarker; DEXA, dual-energy X-ray absorptiometry; DSQ, Dietary Screener Questionnaire; FFQ, Food Frequency Questionnaire; FVs, fruits and vegetables; HC, hip circumference; HEI, Healthy Eating Index; HRQOL, health-related quality of life; hs-CRP, high-sensitivity C-reactive protein; IL-10, interleukin (IL)-10, a pro-inflammatory biomarker; NCI, National Cancer Institute; NHANES, National Health and Nutrition Examination Survey; NR, not reported; NS, not statistically significant; PA, physical activity; RAND-36, survey instrument developed by RAND Corporation to measure HRQOL; RCT, randomized controlled trial; SF-36, Short-Form Health Survey; WC, waist circumference; PAQ, Physical Activity Questionnaire. ^a^ Information obtained from entry in ClinicalTrials.gov. ^b^ Information obtained from personal communication. ^c^ Information not available for intervention effect. ^d^ Results for total sample and not for only the Latino/a subgroup. ^e^ The RAND-36 contains the same items as the SF-36 but uses a different scoring compared to the SF-36. ^f^ Results available for acculturation and outcomes of weight, diet quality, and PA. Mixed evidence for associations of acculturation with body mass index. Study reported statistically significant association for greater acculturation with lower dietary quality, measured with HEI scores, for example, HEI-2015 total scores (67.8 vs. 72.5 out of 100 maximum points, *p* = 0.0009). No significant associations by acculturation with total minutes of moderate-to-vigorous PA, but statistically significant associations for acculturation with leisure PA [46].

**Table 4:** Nutrition interventions for Latino/a cancer survivors: effects on biomarkers.

Intervention Name	Assessment	Timing	Biomarkers for Dietary Intake of Fruits and Vegetables	Biomarkers of Inflammation, Metabolism, Lipids, or Cancer Recurrence	Effects
**Avanzando Juntas/Moving Forward** [36]	Blood sample.	Collected at baseline, post-test (4 months). No follow-up.	N/A	Inflammatory and metabolic biomarkers: cholesterol, lipid, HbA1c adiponectin, leptin, C-peptide, hs-CRP, and insulin resistance measured with glucose, glycogen, and insulin. ^a^	NR ^b^
**Bronx Oncology Living Daily (BOLD) Healthy Living** [37]	N/A	N/A	N/A	N/A	N/A
**Cocinar para Su Salud/Cook for Your Health/Life** [38,39,40]	Blood sample (fasting). DNA sample.	Collected at baseline and 3, 6, and 12 months.	Serum carotenoids: total carotenoids, lutein, α-carotene, beta-carotene, beta-cryptoxanthin, and retinol.	Inflammatory biomarkers: IL-1α, IL-6, IL-8, IL-10, hs-CRP, GM-CSF, and TNF-α.	Increased total carotenoids and serum lutein in the intervention group. Non-significant decreases in inflammatory biomarkers: IL-1α, IL-6, IL-10, TNF-α, and hs-CRPs in the intervention group at 6 months. Borderline increased global DNA methylation in the intervention group.
**La Vida Activa/An Active Life** ^c^ [41]	Blood sample (fasting morning blood draw).	Collected at baseline and 3, 6, 9, and 12 months.	N/A	Inflammatory and metabolic biomarkers: serum cholesterol (total, high-density lipoprotein, indirect low-density lipoprotein), triglycerides, glucose, hs-CRP, insulin, adiponectin, IGF-I, total IGF binding protein-1, IGF binding protein-3, and HOMA-IR.	No effects on metabolic biomarkers using intent-to-treat analysis. Fat loss ≥ 2% decreased insulin, glucose, and HOMA-IR at 6 months. Weight loss ≥ 5% increased IGF-1 BP1 and decreased glucose at 6 and 12 months.
**LIVES (Lifestyle Intervention for Ovarian Cancer Enhanced Survival)** ^c^ [42,43]	Blood sample. DNA sample. Tissue collection of tumor tissue for pathology report.	Collected at baseline and 6, 12, and 24 months.	Plasma carotenoids.	Metabolic, lipid, and mechanistic biomarkers modified by diet and physical activity: insulin glucose, lipids, and telomere length. Prognostic biomarkers: IL-6, and omentin.	NR ^b^
**Mi Vida Saludable/My Healthy Life** [44,45,46]	Blood sample (optional, fasting blood draw).	Collected at baseline and 6 and 12 months.	Plasma carotenoid and tocopherol concentrations.	Inflammatory biomarkers: hs-CRP, IL-6, TNF-α), oxidative stress (e.g., isoprostane). DNA methylation. Metabolic biomarkers: insulin, glucose, insulin growth factor (IGF), and lipid panel.	NR ^b^
**My Health** [47]	N/A	N/A	N/A	N/A	N/A
**Nuestra Salud/For Your Health** [48]	N/A	N/A	N/A	N/A	N/A
**Prescription (Rx) for Better Breast Health** ^c^ [49,50]	Blood sample (12-h fasting).	Collected at baseline and 6 and 12 months.	None	Pro- and anti-inflammatory biomarkers: IL-3, IL-6, IL-8, and IL-10; CRP; and TNFα. Circulating ASCs. Lipid biomarkers: total cholesterol, triglycerides, LDL, and HDL. Metabolic biomarker: HbA1c.	NR ^b^
**Women’s Health Eating and Living Study (WHEL)** [51,52]	Blood draw (fasting). Interviews with review of medical records; tissue collection of tumor tissue for pathology report.	Baseline, year 1, and at end of year 4 (racial/ethnic subgroup analysis).	Plasma carotenoids (β-carotene, α-carotene, lutein/zeaxanthin, lycopene, and β-cryptoxanthin).	None	No effects on cancer recurrence or mortality rates by racial/ethnic group. NR: Effects on carotenoids as biomarker. ^b^

Research assistants extracted information for studies. Effects on biomarkers were defined based on statistically significant effects. The *p*-values for statistical significance varied by study. DEXA, dual-energy X-ray absorptiometry; DNA, deoxyribonucleic acid; FFQ, Food Frequency Questionnaire; GM-CSF, granulocyte-macrophage colony-stimulating factor; HbA1c, hemoglobin A1c; HOMA-IR, homeostasis model assessment for insulin resistance; HDL, high-density lipoprotein; hs-CRP, high-sensitivity C-reactive protein; IGF, insulin-like growth factor; IL, interleukin; LDL, low-density lipoprotein; LIVES, Lifestyle Intervention in Ovarian Cancer Enhanced Survival; N/A, not applicable; NR, not reported; TNF-α, tumor necrosis factor alpha; WC, waist circumference. ^a^ Information obtained from entry in ClinicalTrials.gov. ^b^ No information available for effects. ^c^ Results for total sample and not for only the Latino/a subgroup.

## Data Availability

Data sharing not applicable.

## Supplementary Materials

The following supporting information can be downloaded at: https://www.mdpi.com/article/10.3390/nu15234963/s1, Table S1: Search strategy for additional sources of trials; Table S2: Eligibility criteria for the population, intervention, comparison, outcome, and study design (PICOS); Table S3: Ongoing, future planned, or terminated trials that were not included in this scoping review; Table S4: Attrition, attendance, and adherence for nutrition interventions with Latino/a cancer survivors.
