# Supplementary material for: A Scoping Review to Explore the Potential Benefits of Nutrition Interventions for Latino/a Adult Cancer Survivors in the US
Source: Nutrients. 2023 Nov 29;15(23):4963. doi: 10.3390/nu15234963 (PMC10708293; doi:10.3390/nu15234963)
Supplement: Supplementary file 1 [file nutrients-15-04963-s001.zip › nutrients-2687503-supplementary.pdf]

Supplementary Materials

Table S1: Search strategy for additional sources of trials

Literature Source	Search strategy	Number of records identified and screened	Number excluded	Number of potentially relevant interventions for Latino/a cancer survivors	Number of interventions added to review
ClinicalTrials.gov [30]	Condition or disease: Cancer survivor; Other terms: Hispanic; Intervention/treatment: Diet, dietary, nutrition;[a] Recruiting: All types; Country: U.S.	6[b]	3 excluded trials because no results available. 3 potentially relevant trials. (See Table S3 for ongoing trials)	1 terminated trial Mi Vida Saludable en el Valle (NCT04081298)<br><br>2 completed trials Cocinar para Su Salud (NCT01414062) La Vida Activa. Curves (NCT00811824)	0
Reporter.NIH.gov [31]	Keywords: Nutrition diet dietary intervention Hispanic Latino cancer survivor. No other filters or limits.	32b	30 excluded. 20 duplicates. Of the 12 non-duplicates, 6 studies were excluded: 4 observational studies, and 2	2 completed trials Avanzando Juntas (NCT04321135) Cocinar para Su Salud (NCT01414062)	1 additional trial Avanzando Juntas (NCT04321135)

			research centers. 6 potentially related trials. Excluded 4 trials because they were incomplete or not related to this scoping review. 2 potentially related trials.<br>2 incomplete and relevant trials (Table S2).<br>COACH<br>TEAL<br>2 trials irrelevant for this scooping review<br>Every Day Counts<br>Men Moving Forward		
Cochrane CENTRAL [32]	Title Abstract Keyword: nutrition diet dietary intervention Hispanic Latino cancer survivor.	5	2 excluded trials because both for Cocinar para Su Salud. 3	3 completed trials Cocinar para Su Salud (NCT01414062)	0

	No other filters or limits.		potentially relevant trials.	La Vida Activa. Curves (NCT00811824) Prescription (Rx) for Better Breast Health (NCT02279303)	

This table shows the search strategy for websites for clinical trials, including ClinicalTrials.gov [30], Reporter.NIH.gov [31], and Cochrane Central Register of Controlled Trials (CENTRAL) [32]. Sources were searched originally on January 28, 2023, and again on September 28, 2023 to identify any new trials. Of the 43 records, there were 24 duplicates across websites. Records were excluded for the following reasons: observational studies (n=4), not research studies (n=2), and interventions without Latino/a cancer survivors (n=2). Of the seven interventions relevant for Latino/a cancer survivors, one trial was terminated, four trials were completed, and three trials were ongoing and would be starting soon. Ongoing and future trials are shown in Table S3. One new intervention was added from the website search.
NCT: National Clinical Trial; NIH: National Institutes of Health. [a] ClinicalTrials.gov search was also completed using "dietary intervention" for intervention/treatment (n = 8 results). However, the results were the same as the previous search. The two additional results were activity-focused interventions without a nutrition or diet focus. [b] In the updated search from September 28, 2023, there were additional records identified from the search of websites, but none met eligibility criteria. For example, from ClinicalTrials.gov, there was one additional study from September 28, 2023, but this new trial is not yet recruiting. See Table S3.

Table S2: Eligibility criteria for the population, intervention, comparison, Outcome, and Study design (PICOS)

Component	Inclusion Criteria	Exclusion Criteria
Population	Samples with (human) adults who were diagnosed with cancer and self-identifying as Hispanic or Latino/a and living in the U.S.	Samples with Latino/a adults who were diagnosed as children or cancer survivors from other racial/ethnic backgrounds.
Intervention	Any intervention or program with a nutrition education, including experiential education like cooking classes, tastings, or field trips, behavioral nutrition, or dietary change/dietary modification component or activity.	Intervention or program without a nutrition component.
Comparison	None	None
Outcome	Any outcomes relevant to nutritional status for cancer survivors including but not limited t cancer recurrence, diet, eating behaviors, body composition or weight, well-being, quality of life, sleep, stress, physical activity, inflammation, adverse cardiometabolic health, pain.	Physical activity only; Outcomes not relevant to nutritional status.
Study Design	Experimental study designs, including randomized and non-randomized designs.	Observational studies.

Table S3: Ongoing, future planned, or terminated trials that were not included in this scoping review

Intervention Name (NCT Number)	Setting	Sample	Intervention Focus	Study Design (Duration)	Status
SMLI with Hispanic Cancer Survivors and Caregivers; Official name: Symptom Management and Lifestyle Intervention with Hispanic Cancer Survivors and Caregivers [64] (NCT05364372)<br><br>Note: Trial with the Vida Plena study [62,63].[a]	Tucson, AZ<br><br>Southern AZ Recruitment from U.S.-Mexico border community	Female Hispanic cancer survivors who finished curative intent treatment for cancer within the last 12 months. English or Spanish speakers	Lifestyle (Nutrition and PA)	RCT (12 weeks)	Recruiting
The COACH Study: A Framework for Individualized Coaching in Young Adult Cancer Survivors to Encourage Healthy Behaviors; Official name: The "COACH" Study: Individualized COaching in Young Adult Cancer Survivors to Encourage Healthy Behaviors [65] (NCT05434702)	Tampa, FL	Minority young adult (YA) cancer survivors, diagnosed between 19 - 29 years old who finished treatment within the last 12 months.<br><br>English speakers only	Lifestyle (Nutrition and PA)	RCT (NR)	Recruiting
Remotely Delivered, Culturally Tailored Weight Loss Interventions Among	Multi-site study conducted	Spanish and/or English	Lifestyle (Nutrition and PA)	RCT (12 months)	Not yet recruiting

Latina Breast Cancer Survivors (The ¡Vida! Program);<br>Official name: Using a SMART Design to Evaluate Remotely Delivered, Culturally Tailored Weight Loss Interventions among Latina Breast Cancer Survivors [66] (NCT05930483)	in Seattle, WA				
Trial of Exercise and Lifestyle in Women with Ovarian Cancer (TEAL);<br>Official name: Trial of Exercise and Lifestyle (TEAL) in Women with Ovarian Cancer [67] (NCT05761561)	Multi-site study conducted in New Haven, NJ and Miami, FL	Female ovarian cancer survivors who are scheduled for treatment. No mention of race/ethnicity of participants.<br><br>Spanish or English speakers	Medical nutrition (Nutrition and PA)	RCT (18 weeks on average, depending on length of chemotherapy)	Not yet recruiting
eHealth Diet and Physical Activity Program for the Improvement of Health in Rural Latino Cancer Survivors (MiVSEEV);<br>Official name: Adaptation and Evaluation of an Online and eHealth	Seattle, WA Recruitment from the Lower Yakima Valley	Latino adults with history of cancer and chronic disease, at least 2 months-post treatment. Spanish speakers or	Culturally-based lifestyle (Nutrition and PA)	RCT<br>(3 months)	Terminated due to slow accrual

Diet and Physical Activity Program to Improve Cardiometabolic Health in Rural Latino Adults "Mi Vida Saludable en el Valle" [79] (NCT04081298)		bilingual (Spanish and English)			

This table shows a list of potentially relevant nutrition interventions for Latino/a cancer survivors, which were not included, because the trials were in the design phase, ongoing, or there were no publications available at the time. Trials were identified from website search. Intervention name is the official title posted to ClinicalTrials.gov [30].
AZ: Arizona; CBPR: Community-based participatory research; FL: Florida; NCT: National Clinical Trial; NIH: National Institutes of Health; NJ: New Jersey; PA: Physical activity; RCT: Randomized controlled trial; WA: Washington.
[a]Information obtained via personal correspondence.

Table S4: Attrition, attendance, and adherence for nutrition interventions with Latino/a cancer survivors

	Intervention Name	Attrition	Attendance	Adherence
1	Avanzando Juntas/Moving Forward [36]	NR	NR	NR
2	Bronx Oncology Living Daily (BOLD) Healthy Living [37]	48.2% at 4 or 12-week post-test† (n = 40 complete pre/post anthropometric measures, 83 total)	62.5% attended half of sessions (median attendance).[a]	NR
3	Cocinar Para Su Salud/Cook for Your Health/Life [38–40)	13% at 6 months† (87% retention); 17% at 12 months.	38% attended all 9 sessions; 26% attended 7-8 sessions; 18% 1-6 sessions; 18% did not attend any sessions.	NR
4	La Vida Activa/An Active Life [41]	9.5% at 12 months† (90.5% retention)	Average attendance of 5.1 versus 5.0 out of 6 sessions for the intervention versus the waitlist control, respectively.	Assessed adherence index for behavioral targets based on changes in food frequency questionnaire and dietary behaviors at baseline, month 6 and 12. Intervention group participants had a much higher adherence index score at month 6

	Intervention Name	Attrition	Attendance	Adherence
				and they mostly maintained score at month 12.
5	LIVES (Lifestyle Intervention for Ovarian Cancer Enhanced Survival) [42,43]	11.5% at 24 months† (88.5% retention)	Attendance for coaching calls was 84.8% Ş (28 out of 33 calls in intervention) versus 86.4% Ş (19 out of 22 calls in control) over 24-month period.	Assessed lifestyle behavior goal adherence based on a score, created with data from study participation and behavior changes. Results for adherence: NR.
6	Mi Vida Saludable/My Healthy Life [44–46]	NR	NR	NR
7	My Health [47]	8.8%† (n = 7 lost to follow-up out of 80).	NR	NR
8	Nuestra Salud/For Your Health [48]	49% at 12 weeks† (Lost 23 dyads out of 45 enrolled)	For dyads who started the intervention, 86% (n = 20) completed ≥75% coaching sessions. For Latino/a cancer survivors: ≥80% completed all 12 sessions; on average, cancer survivors completed 9.3 out of 12	Assessed lifestyle behavior goal attainment in the intervention group only, based on scores from lifestyle behaviors. Cancer survivors reported medium to large effects for total fruits and vegetables, vegetables, and

	Intervention Name	Attrition	Attendance	Adherence
			coaching. sessions.	sugar intake at post-test.
9	Prescription (Rx) for Better Breast Health [49,50]	18.3% at 6 months	NR	Assessed adherence to Mediterranean diet based on 14 questions. For both intervention and control groups, there was low adherence to Mediterranean diet guidelines. However, relative to control group, intervention group increased significantly for adherence for three guidelines: red meat, fish or shellfish, and commercially-prepared sweets or pastries ($p < 0.001$).
10	Women's Healthy Eating and Living Study (WHEL) [51,52]	Results for adherence for Latino/a subgroup: NR	Results for adherence for Latino/a subgroup: NR	Developed WHEL Adherence Score to measure adherence to study's behavioral targets and assessed at

Intervention Name	Attrition	Attendance	Adherence
			different time points. Results for adherence for Latino/a subgroup: NR.

Research assistants extracted information for studies from published abstracts and journal articles, except when indicated by footnotes. Study dates are for the enrollment or intervention period described in the original research record or available from ClinicalTrials.gov or Reporter.NIH.gov websites accessed on 28 September 2023. Loss to follow or attrition was calculated based on retention or attrition between enrollment and outcome assessment, as reported by each study. Attendance was for intervention contacts. Adherence assessment varied by study. [a] Intervention had aimed for 75-80% attendance, but because they did not achieve this goal, the study reported attendance for proportion of the sample achieving at least 60% attendance. 67.3% of participants in the 12-week programs achieved ≥60% attendance, compared to 41.9% in the 4-week programs [37].

[†] Calculated for this review based on published data.
